# Maximum entropy relaxation of multi-way cardinality constraints for synthetic population generation

**DOI:** 10.3389/frai.2026.1840945

**Published:** 2026-10-23

**Authors:** François Pachet, Jean-Daniel Zucker

**Affiliations:** 1 ImagineAllThePeople, Paris, France; 2 Unité de Modélisation Mathématique et Informatique des Systémes Complexes (UMMISCO), Institut de Recherche pour le Développement (IRD), Sorbonne Université, Paris, France

**Keywords:** cardinality constraints, constraint programming, exponential family models, maximum entropy, synthetic population generation

## Abstract

Generating synthetic populations from aggregate statistics is a core component of microsimulation, agent-based modeling, policy analysis, and privacy-preserving data release. Beyond classical census marginals, many applications require matching heterogeneous unary, binary, and more generally multi-way constraints derived from surveys, expert knowledge, or automatically extracted descriptions. Constructing populations that satisfy such multi-way constraints simultaneously poses a significant computational challenge. We consider populations where each individual is described by categorical attributes and the target is a collection of global frequency constraints over attribute combinations. Exact formulations scale poorly as the number and arity of constraints increase, especially when the constraints are numerous and overlapping. We propose a maximum-entropy relaxation of this problem. Multi-way cardinality constraints are matched in expectation rather than exactly, yielding an exponential-family distribution over complete population assignments and a convex optimization problem over Lagrange multipliers. The convex program defines the theoretical MaxEnt solution; in practice we approximate it with a fixed-budget MCMC moment-matching procedure, and all reported results measure the empirical performance of this finite-budget approximation. We evaluate the approach on NPORS-derived scaling benchmarks with four to 40 attributes, and on a controlled synthetic benchmark family generated independently of NPORS, and compare it primarily against generalized raking. The results show that MaxEnt becomes increasingly advantageous as the number of attributes and ternary interactions grows, while raking remains competitive on smaller, lower-arity instances.

## 1 Introduction

Synthetic population generation has become a foundational problem for contemporary AI systems in settings where individual-level microdata are unavailable, legally restricted, or prohibitively expensive to collect. At the same time, many downstream tasks such as simulation, forecasting, and counterfactual analysis, require large numbers of representative agents rather than aggregate statistics alone.

This need arises across a growing range of domains. In public policy and politics, synthetic populations are used to evaluate reforms, study voting behavior, and explore demographic scenarios without exposing sensitive personal data. In health and epidemiology, they support large-scale simulations of disease propagation, intervention strategies, and healthcare demand. In retail and marketing, they enable customer behavior modeling, demand forecasting, and what-if analyses in environments where individual data are fragmented or protected. More broadly, synthetic populations provide a bridge between aggregate statistics, expert knowledge, and agent-based models, allowing AI systems to reason about societies, markets, and institutions at scale while respecting privacy and regulatory constraints. Recent work on LLM-based synthetic personas further broadens this perspective beyond classical microsimulation: DeepMind's *Persona Generators* expands compact contextual descriptions into diverse synthetic populations tailored to arbitrary settings, with an explicit emphasis on covering rare but consequential trait combinations (Paglieri et al., 2026). This renewed interest also aligns with a broader practitioner view that synthetic data can serve as a flexible design lever for shaping AI system behavior^1^.

In these settings, individuals are described by a vector of categorical attributes (e.g., age bracket, gender, region, occupation), and the population must match a set of published or estimated aggregates. A convenient formalization is to specify a set of frequency constraints over *patterns* of attribute values. For example: “52% female” (unary), “18% female and age 35–44” (binary), or “4% female, age 35–44, and living in Île-de-France” (ternary). Such requirements are naturally expressed in constraint programming as cardinality constraints (Rossi et al., 2006). Unary cardinalities are efficiently handled by the Global Cardinality Constraint (GCC) with strong filtering (Régin, 1996). However, multi-attribute constraints (arity ≥2) are typically modeled as *Sum* constraints over reified predicates or over contingency-table cell counts. These overlapping linear constraints often provide weak propagation and become difficult to satisfy at scale, especially when many higher-order constraints are specified or when targets are noisy or mutually inconsistent. Note that these constraints are imprecise in nature, which calls for approximate rather than exact methods.

Synthetic population generation has been extensively studied in microsimulation, transportation modeling, and survey methodology, using approaches ranging from combinatorial optimization and calibration to iterative reweighting and probabilistic modeling (Beckman et al., 1996; Voas and Williamson, 2000; Deming and Stephan, 1940; Fienberg, 1980). These methods have enabled large-scale applications, but typically rely on either unary marginals or fully specified contingency tables, and assume internal consistency of the target statistics. In contrast, many contemporary applications involve selectively specified, heterogeneous, and potentially noisy multi-way constraints, for which existing approaches either do not scale or fail to provide robust solutions. This gap motivates alternative formulations that can accommodate partial and higher-order information without requiring exact feasibility.

This paper proposes a probabilistic alternative: a maximum-entropy relaxation of multi-way cardinality-constrained population synthesis. Instead of searching directly for a discrete population, we infer a distribution over full attribute assignments whose expectations match the required frequencies. By the maximum-entropy principle (Jaynes, 1957), the solution belongs to an exponential family with one parameter per constraint.

This transforms a difficult combinatorial problem into convex optimization over Lagrange multipliers, after which populations can be sampled efficiently. By construction, the maximum-entropy distribution is as unbiased as possible given the imposed constraints.

### 1.1 Contributions

We provide: (i) a formalization of population synthesis constraints as multi-way cardinality (Sum) CSPs over contingency tables; (ii) a maximum-entropy relaxation that recasts this problem as convex optimization over an exponential-family model; (iii) a practical MCMC-based moment-matching procedure for fitting and sampling that scales to tens of attributes without enumerating the joint space, together with a convex penalized extension for inconsistent targets; and (iv) an NPORS-derived benchmark family that reveals when MaxEnt overtakes generalized raking as dimensionality and constraint arity increase.

## 2 Related work

### 2.1 Cardinality constraints in CP

The Global Cardinality Constraint (GCC) (Régin, 1996) enforces lower and upper bounds on the number of occurrences of atomic values across a set of variables sharing a domain, and supports strong propagation via flow-based filtering. In population synthesis, unary marginals can be expressed directly as GCCs over per-individual attribute variables. However, GCCs do not naturally extend to multi-attribute patterns: binary and ternary marginals correspond to joint counts rather than counts of atomic values, and therefore cannot be captured by a single GCC over the original variables. Such constraints typically require reification and additional Sum constraints, or contingency-table formulations with explicit count variables, which substantially weakens propagation. Recent work by Petit and Pachot (2025) proposes a CP framework for exact synthetic population generation from aggregate statistics and structural relations, using a batch-based construction strategy rather than a single global solve. The paper provides a limited extension-preserving result for the marginal-distribution objective in isolation, but it does not prove global completeness or global optimality for the full model once horizontal constraints, diversity, joint features, time limits, and irrevocable batch commitments are included. Our work targets the same Sum-based regime, but replaces exact feasibility by a probabilistic relaxation and optimizes a single global model over all constraints at once; it should therefore be viewed as complementary to these encodings rather than as a replacement for classical GCC propagation.

### 2.2 Contingency tables, IPF, and log-linear models

Fitting contingency tables from marginal distributions has a long tradition in statistics, notably through iterative proportional fitting (IPF) and log-linear models (Deming and Stephan, 1940; Bishop, 1967; Fienberg, 1980). When only first-order marginals are specified, IPF is known to converge to the unique maximum-entropy distribution consistent with these constraints, and can be viewed as a special case of maximum-likelihood estimation in log-linear models with main effects only (Csiszár, 1975; Darroch and Ratcliff, 1972).

This equivalence extends in principle to higher-order margins: iterative proportional fitting over the full contingency table converges to the maximum-entropy distribution consistent with any compatible set of multi-way margins (Darroch and Ratcliff, 1972; Csiszár, 1975). What breaks down at scale is representation, not principle. Enforcing binary or ternary marginals using IPF then requires operating on full contingency tables whose dimensionality grows exponentially with the number of attributes (Fienberg, 1980). In realistic population synthesis settings, only a sparse and heterogeneous subset of multi-way marginals is available, which prevents the construction of such tables in practice (Voas and Williamson, 2000; Lomax and Norman, 2016).

Several extensions of raking have been proposed to incorporate additional constraints, including generalized regression estimators and iterative proportional updating (IPU) (Deville and Särndal, 1992; Ye et al., 2009). These approaches rely on heuristic weight adjustments and auxiliary interaction variables, and do not provide general guarantees of feasibility or convergence when constraints are overlapping or selectively specified (Chambers and Skinner, 2003; Lomax and Norman, 2016). IPF, raking, and calibration estimators nonetheless remain the dominant and often preferred tools for population synthesis, and we adopt generalized raking as our central empirical baseline throughout this paper. Their difficulty is not a lack of principle but a mismatch of design: they are built to reproduce a consistent set of margins, and offer limited control when the required contingency information is incomplete, high-dimensional, or sparse. In that regime they can reproduce the imposed margins while providing little guidance on rare or unobserved higher-order combinations, and sequential reweighting can become unstable when the selected margins overlap and are not mutually consistent after rescaling to a finite population. We therefore position the maximum-entropy formulation as addressing these practical limitations, extending the IPF/raking family to overlapping multi-way constraints, rather than as a replacement for it.

### 2.3 Bayesian networks, probabilistic graphical, and deep generative models

Bayesian networks represent joint distributions through local conditional dependencies structured as directed acyclic graphs (Pearl, 1988), and have been used directly for population synthesis by learning a factorized joint from microdata and sampling from it (Sun and Erath, 2015). While effective for probabilistic inference under assumed causal or dependency structures, they are not well suited for enforcing exact or near-exact global cardinality constraints over multi-variable configurations. Encoding such constraints within a Bayesian network would require auxiliary counting variables or high-order dependencies spanning the entire population, thereby eliminating the tractability advantages of Bayesian factorization and rendering inference computationally prohibitive (Cooper, 1990).

More broadly, a large family of deep generative models now targets high-dimensional tabular and population-like data. Variational autoencoders scale population synthesis to many attributes and can generate micro-agents beyond the observed sample (Borysov et al., 2019); conditional GANs model mixed-type tabular data (Xu et al., 2019); and deep generative models have been applied specifically to recover rare and unobserved feature combinations (sampling zeros) that reweighting methods cannot introduce (Garrido et al., 2020). Diffusion models (Kotelnikov et al., 2023) and transformer or language-model backbones (Borisov et al., 2023; Solatorio and Dupriez, 2023) further improve the realism of synthetic tabular records, and large language models have recently been used directly for feasible and diverse population synthesis (Lim et al., 2025), connecting to the LLM-based synthetic-persona work discussed in the introduction (Paglieri et al., 2026).

These approaches are complementary to ours rather than competing. They are density estimators or microdata generators trained on individual-level records, and they excel at reproducing complex dependencies and, in some cases, at extrapolating to rare combinations; our setting instead fits a distribution directly from aggregate multi-way cardinality constraints, without access to microdata, and makes the constraint residuals explicit and auditable. When microdata are available, the two are naturally combined, with a generative model supplying a prior or a plausible support and the maximum-entropy layer enforcing the aggregate margins. Moreover, Bayesian networks and most generative models do not guarantee the preservation of observed marginal distributions, whereas maximum-entropy models explicitly enforce aggregate constraints through convex optimization. The approach proposed here is therefore better understood as a constraint-driven distribution construction method rather than as a probabilistic inference problem amenable to standard Bayesian network formulations.

### 2.4 Positioning our work

Our approach directly targets the regime where constraints are expressed as sums over arbitrary attribute patterns, including unary, binary, and ternary marginals. Rather than enumerating full contingency tables or relying on heuristic reweighting schemes, we formulate population synthesis as a maximum-entropy problem under linear expectation constraints.

In this perspective, full-table IPF solves the same maximum-entropy problem whenever the joint contingency table is explicitly representable (Table 1); our contribution targets the regime where it is not: selectively specified, overlapping multi-way margins over a joint space that cannot be enumerated. Our contribution should therefore be viewed as extending classical population synthesis techniques to the Sum-based constraint regime that arises in realistic high-dimensional settings. A terminological convention applies throughout: “the MaxEnt solution” refers to the mathematical optimum of the convex program of Section 4, while every reported population is produced by the finite-budget MCMC approximation of Section 5; the empirical sections measure the performance of that procedure, not properties of the exact optimum.

**Table 1:** Taxonomy of approaches: required input, treatment of selectively specified overlapping multi-way margins, guarantee, and role in this study.

Family	Required input	Overlapping multi-way margins	Guarantee	Role in this study
Raking, calibration, IPU	Aggregate margins (IPU: seed sample)	Sequential per-margin reweighting; updates can interfere	Approximate weights	main baseline (generalized raking)
Full-table IPF	Margins + representable joint table	Exact fit of compatible margins, in principle	Converges on consistent margins	Exact-table check at *K* ≤ 12
Exact combinatorial (CP-SAT, ILP)	Aggregate margins	Exact cell-count encoding	Optimality certificates when solved	Exact reference (Figure 1); small cases within the tested budget
Bayesian networks, microdata models	Individual-level microdata or expert structure	Not consumed as constraints	Model-based	Discussed; different input regime
Deep generative (VAE, CTGAN, diffusion, LLM-based)	Individual-level records	Not consumed as constraints	None on margins	Discussed; not like-for-like
MaxEnt relaxation (this paper)	Aggregate overlapping multi-way margins	Single global convex objective	Convex optimum, finite-budget MCMC approximation	Proposed method

Methods that require individual-level microdata solve a different input problem and are therefore discussed rather than run as baselines. Full-table IPF is representable only on the small instances: the joint table would hold 9 × 1015 cells at *K* = 28 and 2 × 1026 at *K* = 40.

## 3 Problem formulation

### 3.1 Attribute space and populations

Let *K* be the number of categorical attributes, with finite domains *D*_1_, …, *D*_*K*_. A persona is a full assignment


$$x=\left(a_{1},\ldots ,a_{K}\right)\in X≜D_{1}\times \cdots \times D_{K},$$

and $\left|X|=\prod_{k=1}^{K}|D_{k}\right|$. A population of size *N* can be represented either as a multiset of individuals {*x*^(1)^, …, *x*^(*N*)^} or, equivalently, as a contingency table of nonnegative integer counts


$$C={\left(C_{x}\right)}_{x\in X},\;C_{x}\in {\mathbb{Z}}_{\geq 0},\;\underset{x\in X}{\sum }C_{x}=N.$$

### 3.2 Multi-Way Cardinality Constraints (Sum-CSP)

We consider constraints specified over *patterns* (subsets) $S_{j}\subseteq X$ with target frequency α_*j*_∈[0, 1] (or target count *M*_*j*_ = α_*j*_*N*). Each pattern induces an indicator feature


$$f_{j}\left(x\right)≜\text{1}\left[x\in S_{j}\right].$$

In a strict CSP over contingency-table variables, the constraints take the form


$$\begin{array}{c} \underset{x\in S_{j}}{\sum }C_{x}=M_{j},\;j=1,\ldots ,m, \end{array}$$ (1)

together with non-negativity and the total-size constraint. When $S_{j}$ corresponds to fixing 1, 2, or 3 attributes, Equation 1 encodes unary, binary, or ternary marginals respectively. As arity and overlap increase, Equation 1 becomes difficult for CP/MIP solvers, and feasibility may fail under inconsistent targets.

### 3.3 Computational hardness and motivation for relaxation

Problem Equation 1 defines a system of linear equalities over |X| integer variables, where |X| grows exponentially with the number of attributes *K*. Even for moderate *K*, explicitly representing all contingency-table cells is infeasible.

Moreover, the constraints in Equation 1 are highly overlapping: each variable *C*_*x*_ typically participates in many pattern constraints, and the resulting constraint matrix is dense and ill-conditioned. As a consequence, exact feasibility is fragile: small inconsistencies in the target marginals may render the system infeasible.

From a computational perspective, solving Equation 1 exactly amounts to finding a nonnegative integer solution to a large-scale multi-way marginal consistency problem, which is known to be NP-hard in general and intractable for standard CP or MIP solvers beyond small instances. Even when feasibility holds, the space of solutions is typically extremely large, making arbitrary solution selection undesirable.

These limitations motivate the use of relaxed formulations that trade exact satisfaction of constraints for probabilistic or entropic consistency, while preserving the essential structure of the marginal information.

## 4 Maximum entropy relaxation

Maximum entropy modeling under linear expectation constraints is equivalent to exponential-family modeling: the MaxEnt solution admits a canonical exponential form whose sufficient statistics correspond to the constrained features. While exponential-family and energy-based models are typically learned from microdata via likelihood-based objectives (Wainwright and Jordan, 2008), our setting departs from this paradigm by inferring parameters directly from aggregate multi-way cardinality constraints. The resulting model is therefore used as an approximate solver and sampler for large-scale combinatorial population constraints, rather than as a density estimator over observed samples.

The maximum entropy principle provides a principled way to construct probability distributions from partial information, originally introduced in statistical physics to characterize equilibrium distributions under macroscopic constraints (Jaynes, 1957). Its central idea is to select, among all distributions satisfying the known constraints, the one that makes the fewest additional assumptions. Beyond physics, maximum entropy models have been successfully applied in machine learning and structured data modeling to capture higher-order statistical regularities while avoiding overfitting (Sakellariou et al., 2017). In our context, this principle yields a global optimization-based relaxation of multi-way cardinality-constrained population synthesis and suggests a natural penalized extension for noisy or mutually inconsistent targets.

### 4.1 Expectation constraints

We replace the integer table *C* by a distribution *p* over X and enforce constraints in expectation:


$$\begin{array}{c} {𝔼}_{p}\left[f_{j}\right]=\underset{x\in X}{\sum }p\left(x\right)f_{j}\left(x\right)=\alpha_{j},\;j=1,\ldots ,m. \end{array}$$ (2)

This is a convex relaxation of Equation 1, replacing exact counts by expected frequencies.

### 4.2 Soft constraints for inconsistent targets

When the target vector α lies outside the moment polytope generated by the features ${\left\{f_{j}\right\}}_{j=1}^{m}$, the equalities in Equation 2 cannot all be satisfied simultaneously. A natural soft alternative is therefore to optimize directly over distributions:


$$\begin{array}{c} \underset{p\in \Delta \left(X\right)}{\min}-H\left(p\right)+\frac{\beta }{2}\overset{m}{\underset{j=1}{\sum }}w_{j}{\left({𝔼}_{p}\left[f_{j}\right]-\alpha_{j}\right)}^{2}, \end{array}$$ (3)

where β>0 controls the overall trade-off between entropy and constraint fidelity and *w*_*j*_>0 assigns a positive relative fidelity weight to constraint *j*. This objective is convex in *p* because negative entropy is convex and each penalty term is a square of a linear functional. When the hard constraints are feasible and β is large, the solution approaches the standard MaxEnt model. In the present paper, however, the empirical evaluation focuses on the hard-constraint regime induced by empirical marginals extracted from observed populations; the penalized formulation in Equation 3 is included to make the treatment of noisy targets explicit, and Section 6.5 evaluates MaxEnt and the raking baseline on deliberately corrupted targets.

### 4.3 MaxEnt principle and exponential family

Among distributions satisfying Equation 2, we choose the one with maximum Shannon entropy:


$$\underset{p\in \Delta \left(X\right)}{\max}H\left(p\right)\;\text{s.t.}\;{𝔼}_{p}\left[f_{j}\right]=\alpha_{j}\forall j,$$

where $H\left(p\right)=-\underset{x}{\sum }p\left(x\right)\log p\left(x\right)$. By classical duality results (Jaynes, 1957; Wainwright and Jordan, 2008), if the constraint set is feasible, the unique maximizer has the exponential-family form


$$\begin{array}{c} p_{\lambda }\left(x\right)=\frac{1}{Z\left(\lambda \right)}\exp\left(\overset{m}{\underset{j=1}{\sum }}\lambda_{j}f_{j}\left(x\right)\right), \end{array}$$ (4)

where λ∈ℝ^*m*^ are Lagrange multipliers and $Z\left(\lambda \right)=\underset{x}{\sum }\exp\left(\underset{j}{\sum }\lambda_{j}f_{j}\left(x\right)\right)$ is the partition function.

### 4.4 Dual objective and convexity

The dual problem is


$$\begin{array}{c} \underset{\lambda \in {\mathbb{R}}^{m}}{\min}\Phi \left(\lambda \right)≜\log Z\left(\lambda \right)-\overset{m}{\underset{j=1}{\sum }}\lambda_{j}\alpha_{j}. \end{array}$$ (5)

Its gradient is


$$\nabla \Phi \left(\lambda \right)={𝔼}_{p_{\lambda }}\left[f\right]-\alpha ,$$

and the Hessian equals the covariance matrix Cov_*p*_λ__(*f*), implying convexity and, under mild conditions, strict convexity (Wainwright and Jordan, 2008).

Two remarks make the status of these conditions for our feature sets explicit. First, identifiability: with unary, pairwise, and ternary indicator cells, the features are linearly dependent by construction. Whenever all cells of a marginal are retained, the indicators of that marginal sum to one for every *x*, and overlapping marginals induce further dependencies (a fully supported binary marginal sums to each of its unary marginals). The Hessian Cov_*p*_λ__(*f*) is therefore singular on such feature sets, Φ is convex but not strictly convex in λ, and individual multipliers are not identifiable: adding any vector of the null space of the feature matrix leaves *p*_λ_ unchanged. The induced *distribution* remains unique whenever the constraints are feasible; only the *parameterization* is redundant. Our implementation retains all observed cells and simply tolerates this over-complete parameterization: the first-order updates of Section 5 only move λ along directions spanned by the moment residuals, and all reported quantities depend on λ only through *p*_λ_. Second, the penalized formulation Equation 3 admits an exact ridge-regularized dual, which we now derive. For β>0 and *w*_*j*_>0, write *r*_*j*_ = *E*_*p*_[*f*_*j*_]−α_*j*_ for the moment residuals. Each quadratic penalty has the Fenchel representation


$$\frac{\beta w_{j}}{2}r_{j}^{2}=\underset{y_{j}\in \mathbb{R}}{\sup}\{y_{j}r_{j}-\frac{y_{j}^{2}}{2\beta w_{j}}\}.$$

Substituting this representation into Equation 3, exchanging the minimization over *p* with the maximization over *y*, which is licit because Δ(X) is compact and convex and the objective is convex in *p* and concave in *y* (Sion's minimax theorem; see also Wainwright and Jordan (2008)), and minimizing over p∈Δ(X) yields


$$\underset{y\in {\mathbb{R}}^{m}}{\max}\{-\log Z\left(-y\right)-y^{⊤}\alpha -\overset{m}{\underset{j=1}{\sum }}\frac{y_{j}^{2}}{2\beta w_{j}}\}.$$

With the change of variables λ = −*y*, this is equivalently


$$\begin{array}{c} \underset{\lambda \in {\mathbb{R}}^{m}}{\min}\Phi_{\text{soft}}\left(\lambda \right)≜\log Z\left(\lambda \right)-\overset{m}{\underset{j=1}{\sum }}\lambda_{j}\alpha_{j}+\overset{m}{\underset{j=1}{\sum }}\frac{\lambda_{j}^{2}}{2\beta w_{j}}. \end{array}$$ (6)

The first-order optimality condition is *E*_*p*_λ__[*f*_*j*_]−α_*j*_+λ_*j*_/(β*w*_*j*_) = 0, or equivalently λ_*j*_ = −β*w*_*j*_(*E*_*p*_λ__[*f*_*j*_]−α_*j*_): in contrast to the hard-constraint problem, the moment residual need not vanish; it is balanced against the magnitude of the corresponding multiplier. The Hessian of the penalized dual is


$$\nabla^{2}\Phi_{\text{soft}}\left(\lambda \right)={\text{Cov}}_{p_{\lambda }}\left(f\right)+\text{diag}(\frac{1}{\beta w_{1}},\ldots ,\frac{1}{\beta w_{m}}),$$

which is positive definite for β>0 and *w*_*j*_>0, since the covariance is positive semidefinite and the diagonal term is positive definite; the penalized dual is therefore strictly convex in λ regardless of the feature redundancies discussed above. It is also coercive: because the features are bounded indicators, log*Z*(λ)−λ^⊤^α is bounded below by an affine function of λ, while the ridge term grows quadratically, so Φ_soft_(λ) → +∞ as ||λ|| → ∞. A unique finite minimizer therefore exists for feasible and infeasible target vectors alike, in contrast with the hard dual Equation 5, which may fail to possess a finite minimizer when α lies outside the moment polytope and some multipliers diverge toward the boundary. We stress that Equation 6 is not a device for selecting among solutions of the hard dual: it is exactly the optimization problem corresponding to the penalized primal Equation 3. An additional benefit is that strict convexity removes the parameter non-identifiability caused by redundant indicator features: the minimizer is unique. For feasible targets, along redundant parameter directions that leave the induced distribution unchanged, the ridge term selects the unique weighted minimum-norm representative. Section 6.5 evaluates the penalized formulation explicitly.

## 5 Optimization and sampling

### 5.1 Learning λ

We learn λ by minimizing Equation 5 using gradient-based optimization. At each iteration, we generate samples from the current model by running an MCMC scheme with Metropolis updates targeting the Gibbs distribution defined by the current parameters. The empirical moments computed from these sampled populations are then used to update λ. The overall procedure is summarized in Algorithm 1.

Algorithm 1 MaxEnt fitting from multi-way cardinality targets. 
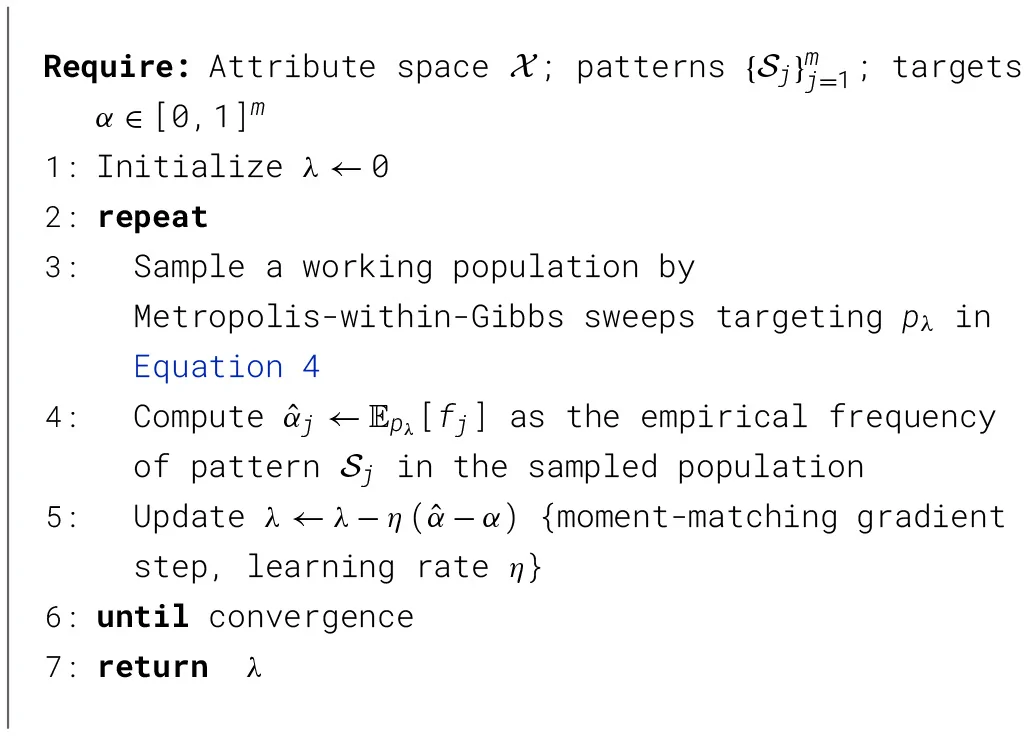

Concretely, the sampler operates on a working population of *N* individuals, initialized uniformly at random over the attribute domains. One sweep visits all *K* variables in a random permutation; for each visited variable, a proposal is drawn simultaneously for every individual as a uniformly random *different* category (a uniform offset among the |*D*_*k*_|−1 alternatives). The move of an individual is accepted with the Metropolis rule: the energy change is $\Delta =\underset{c}{\sum }\left[\lambda_{c}\left(\text{old cell}\right)-\lambda_{c}\left(\text{new cell}\right)\right]$ over the constraints *c* whose scope contains the visited variable, and the move is accepted if Δ ≤ 0, otherwise with probability exp(−Δ). Ten sweeps are interleaved between successive multiplier updates; each update performs a moment-matching gradient step, $\lambda_{c,a}\leftarrow \lambda_{c,a}+\eta \left(M_{c,a}-{\hat{M}}_{c,a}\right)/N$, where *M*_*c, a*_ and ${\hat{M}}_{c,a}$ are the target and current counts of cell *a* of constraint *c*, with initial learning rate η = 0.5 decayed geometrically so that the final rate is about 1% of the initial one. The number of outer iterations is set per instance: roughly one iteration per five constrained cells for the 12-, 20-, and 28-variable benchmarks (246, 565, and 926), while the 4- and 40-variable benchmarks use the default budget of 120; the reported population is drawn after 50 additional sweeps. There is one multiplier per observed marginal cell, so the parameter count equals the constrained-cell count of Table 2 and the joint space X is never enumerated at any point.

**Table 2:** Number of nonzero atomic cells induced by the selected unary, binary, and ternary marginals in the NPORS-derived scaling benchmarks (*N* = 5, 626 in the source population).

Problem	Unary	Binary	Ternary	Total
4-Var	12	54	92	158
12-Var	35	117	1,082	1,234
20-Var	67	510	2,249	2,826
28-Var	107	703	3,824	4,634
40-Var	197	1,329	17,883	19,409

Reported counts correspond to observed support sizes summed over retained marginals, not simply to the number of variables, pairs, or triples. For *K*≥20, the numbers of retained binary and ternary marginals are capped at 50.

### 5.2 Generating a population

Once λ is learned, we sample a population of size *N* i.i.d.:


$$x^{\left(1\right)},\ldots ,x^{\left(N\right)}\sim p_{\lambda }.$$

Empirical frequencies concentrate around targets by standard concentration inequalities. If exact integer marginals are required, a lightweight *repair* step (e.g., local swaps) can be applied to match selected constraints while minimally perturbing others (left for future work).

## 6 Experimental evaluation

We evaluate the proposed Maximum Entropy relaxation on a family of NPORS-derived scaling benchmarks with ternary-or-less cardinality constraints. The goal is to isolate how relative performance changes with the number of attributes, the constraint arity, and the sampled population size.

### 6.1 Experimental setup

To obtain realistic yet controllable instances, we use the 2024 National Public Opinion Reference Survey (NPORS) (Pew Research Center, 2024) as a source population. After preprocessing, the dataset contains *N* = 5, 626 respondents and several hundred categorical or discretized variables with explicit missing-value codes.

We construct five NPORS-derived problems by selecting subsets of *K*∈{4, 12, 20, 28, 40} categorical attributes with moderate domain sizes, clear semantic interpretation, and limited structural missingness. Each subset induces an empirical population in the formalism of Section 3, from which we extract global cardinality constraints over attribute patterns of increasing arity:

- **Unary constraints**, corresponding to marginal distributions of single attributes;
- **Binary constraints**, corresponding to joint marginals over pairs of attributes;
- **Ternary constraints**, corresponding to joint marginals over triples of attributes.

Unary marginals are always included in full. For *K* < 20, all candidate pairs and triples are retained. For *K*≥20, binary and ternary interactions are ranked by informativeness and truncated to the top 50 pairs and top 50 triples, respectively. This keeps the benchmark family comparable across problem sizes while allowing the number of atomic constraints to reflect domain size and interaction complexity.

#### 6.1.1 Inputs

The construction algorithm takes as input:

- a population dataset $C={\left(C_{x}\right)}_{x\in X}$;
- a binary constraint budget $n_{2}\in {\mathbb{N}}^{+}$ or rate ρ_2_∈(0, 1), controlling the number of variable pairs retained;
- a ternary constraint budget $n_{3}\in {\mathbb{N}}^{+}$ or rate ρ_3_∈(0, 1), controlling the number of variable triples retained.

Unary constraints are always extracted in full (one constraint per attribute).

#### 6.1.2 Variable selection by informativeness

Rather than selecting pairs and triples arbitrarily, we rank them by their statistical informativeness and retain the top-*k* most informative ones:

- **Binary pairs** are ranked by *Normalized Mutual Information* (NMI) computed on the joint distribution of each pair in the population;
- **Ternary triples** are ranked by *KL divergence* between the observed joint marginal and the MaxEnt-IPF reference distribution that matches all three pairwise marginals of the triple.

Given a budget *n*_2_ (resp. *n*_3_), the top-*n*_2_ pairs (resp. top-*n*_3_ triples) are retained. When a rate ρ_2_ (resp. ρ_3_) is specified instead, *k* = ⌈ρ × |candidates|⌉ pairs (resp. triples) are selected.

#### 6.1.3 Output

The algorithm outputs a constraint problem defined by a set C of global Sum constraints enforcing exact pattern counts for the selected marginals. The total number of atomic constraints equals the sum of the number of observed values across all selected attributes, pairs, and triples which may be orders of magnitude larger than the number of selected variable pairs or triples (e.g., 50 triples yielding up to 17,883 atomic constraints for a 40-variable problem).

#### 6.1.4 Constraint extraction algorithm

The construction procedure is summarized in Algorithm 2. Unary, binary, and ternary marginals are extracted from the empirical population and included according to the specified budgets or rates.

Algorithm 2 Extraction of reference constraints from a population dataset. 
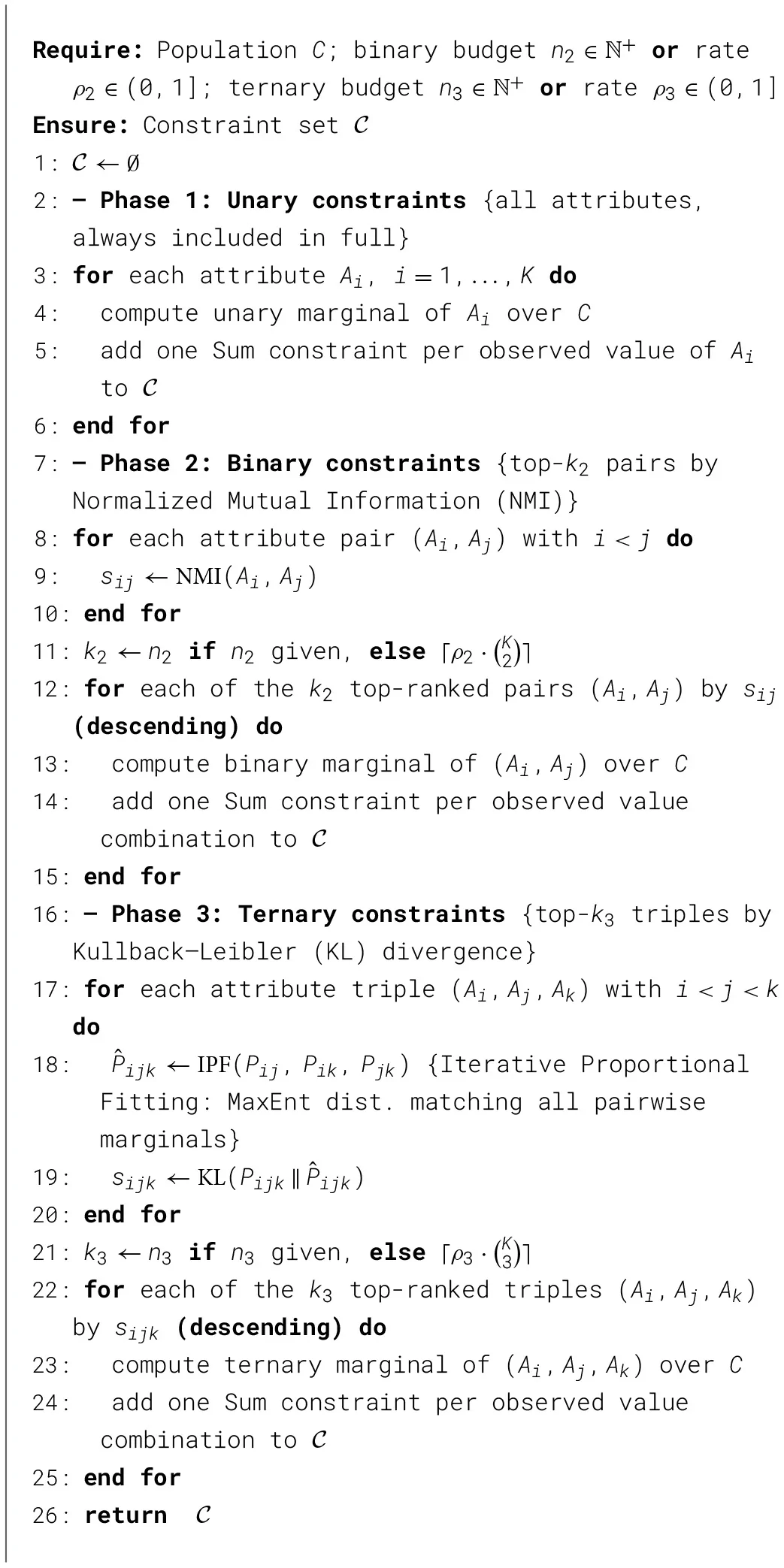

This procedure yields the five NPORS-derived scaling benchmarks used throughout the paper. Table 2 reports the number of atomic constraints, defined as the observed nonzero cells contributed by the retained unary, binary, and ternary marginals, rather than the number of marginals themselves. The reported values are therefore obtained by summing the observed support sizes of all retained marginals within each arity. In this setting, the support size of a marginal is the number of category combinations that occur at least once in the source population. For example, the ternary marginal BORN-DEVICE1A-EMINUSE^2^ involves three variables with three observed levels each, so its full Cartesian product contains 3 × 3 × 3 = 27 possible cells; however, only 22 of these combinations are observed in the NPORS source population, and its support size is therefore 22. The 4-Var benchmark makes this accounting explicit: the four variables contribute 4 × 3 = 12 unary constraints, the six retained pairs each have full 3 × 3 support and thus contribute 6 × 9 = 54 binary constraints, and the four retained triples have support sizes 22, 23, 25, and 22, which sum to 92 ternary constraints. The total for 4-Var is thus 12+54+92 = 158. The same principle explains the larger counts in higher-dimensional benchmarks: for instance, the 12-Var benchmark contains 12 unary marginals but 35 unary atomic constraints, 14 binary marginals but 117 binary atomic constraints, and 44 ternary marginals but 1,082 ternary atomic constraints. More generally, the ternary count grows rapidly with both the number of retained triples and the attribute domain sizes, reaching 17,883 atomic constraints in the 40-Var benchmark despite the cap of 50 retained triples.

#### 6.1.5 Compared methods

The main empirical comparison is between MaxEnt and generalized raking. We focus on this comparison because both methods produce approximate synthetic populations under the same selected marginals and remain meaningful across all benchmark sizes. Exact CP encodings motivate the relaxation but are not used as the primary quantitative baseline in this paper.

We justify this choice of baseline explicitly. Among the families surveyed in the related work, generalized raking is the only widely deployed method that operates in the same input regime as ours: it consumes selectively specified, overlapping multi-way aggregate margins and requires no individual-level microdata, and the IPF/calibration family remains the dominant tool in practical population synthesis (Deming and Stephan, 1940; Deville and Särndal, 1992; Ye et al., 2009). Iterative combinatorial optimization is represented in our study by the exact CP-SAT reference model below, whose scaling limits (Figure 1) are precisely what motivates the relaxation. Deep tabular generators such as VAEs, CTGAN, diffusion models, and language-model-based generators (Borysov et al., 2019; Xu et al., 2019; Kotelnikov et al., 2023; Borisov et al., 2023) are trained on individual-level records and do not take aggregate cardinality constraints as input; they address a different problem setting and are therefore discussed as complementary approaches in the related work rather than run as baselines. Combining such generators with our constraint-driven layer is a natural direction for future work.

**Figure 1:**
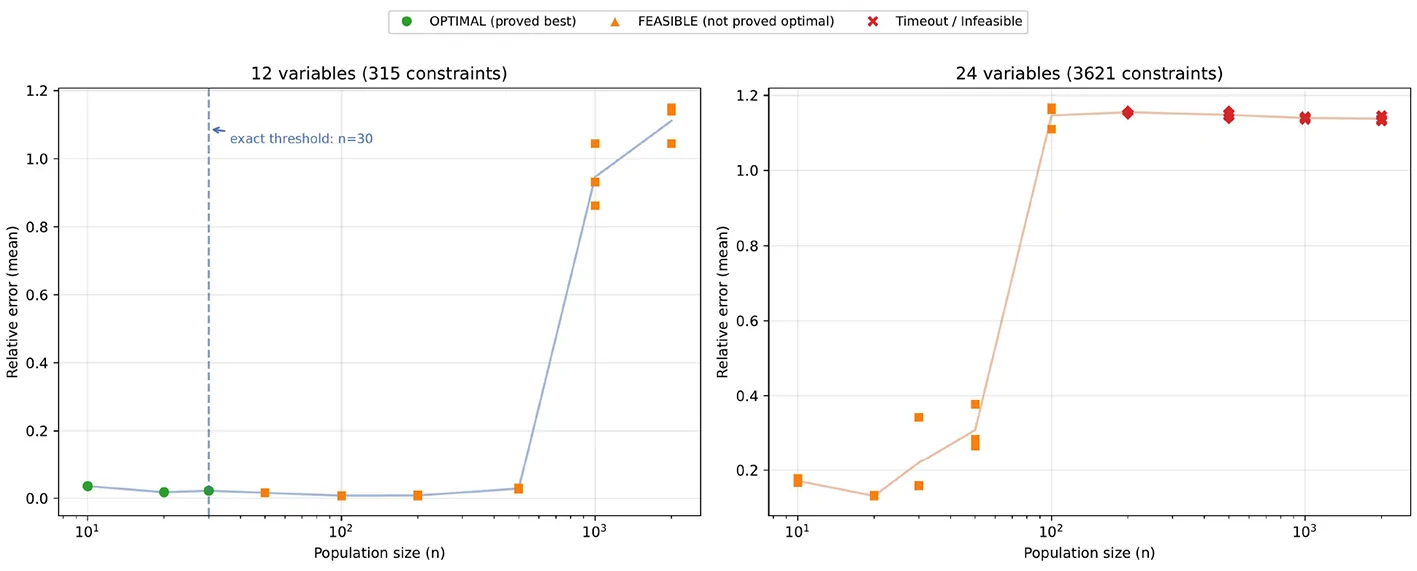
Scalability of the CP-SAT solver. Left: 12 variables (315 target cells); the solver proves optimality up to *n* = 30 (green dots). Right: 24 variables (3,621 target cells); the solver never proves optimality. Markers indicate solver status: optimal (proved best), feasible (not proved optimal), and timeout/infeasible. Error bars show the range over three random seeds. The time limit is 120 s per run.

Table 1 condenses this discussion into an explicit taxonomy: for each family, the input it requires, how it treats selectively specified overlapping multi-way margins, the nature of its guarantee, and its role in this study, so that the comparability (or non-comparability) of each excluded method is stated rather than implied.

#### 6.1.6 OR-tools CP-SAT formulation

For completeness, we also implemented an exact reference model with OR-Tools CP-SAT. Let *V* denote the set of selected categorical variables and let *n* be the desired size of the synthetic population. For each variable *v*∈*V*, the domain $D_{v}$ is the set of categories observed in the reference data, with missing-value codes retained as ordinary categories. From the reference population, we compute all unary marginals and, up to a user-defined maximum arity *k*∈{1, 2, 3}, all pairwise and ternary marginals. For each scope *S*⊆*V* such that 1 ≤ |*S*| ≤ *k*, and for each observed assignment $a\in \prod_{v\in S}D_{v}$, we define a target count *t*_*S, a*_. When the requested synthetic size differs from the source population size, these counts are proportionally rescaled so that they sum to *n*. The CP-SAT model then introduces integer decision variables *x*_*i, v*_ encoding the category assigned to variable *v* for synthetic individual *i*, together with auxiliary count variables


$$y_{S,a}=\overset{n}{\underset{i=1}{\sum }}\text{1}\left[x_{i,S}=a\right]$$

for every stored marginal cell. Depending on the experimental setting, the model either enforces *y*_*S, a*_ = *t*_*S, a*_ exactly (hard mode) or minimizes the ℓ_1_ discrepancy


$$\underset{S,a}{\sum }|y_{S,a}-t_{S,a}|.$$

Only marginal cells with nonzero empirical support are stored in the generated instance; consequently, unobserved cells are not encoded as structural zeros and remain unconstrained. In this paper, this CP-SAT model is included as an exact reference formulation, while the systematic quantitative comparison focuses on MaxEnt and generalized raking.

#### 6.1.7 Implementation details

All MaxEnt models are fitted by MCMC moment matching: starting from a random population of *N* individuals, we alternate Metropolis-within-Gibbs sweeps (10 per outer iteration) with moment-matching gradient updates of the multipliers λ (initial learning rate 0.5 with geometric decay; 120 to 926 outer iterations depending on the instance, see Table 3), and draw the reported population with 50 further sweeps; the joint space X is never enumerated. Generalized raking follows a standard iterative proportional fitting procedure: each individual carries a positive weight, initialized uniformly, and constraints are processed sequentially; for each constraint, the weights of matching individuals are multiplicatively rescaled so that the weighted sum matches the target value. The procedure is iterated until the maximum multiplicative weight change falls below 10^−6^ or a cap of 500 iterations is reached.

**Table 3:** MaxEnt fitting cost on the arity-3 benchmarks (*N* = 5, 626).

Problem	Atomic cells	Iterations	s / outer iter	Est. fit time (s)
4-Var	158	120	0.09	10
12-Var	1,234	246	0.50	123
20-Var	2,826	565	0.93	527
28-Var	4,3634	926	1.11	1,028
40-Var	19,409	120	1.34	161

The per-outer-iteration MCMC cost scales with the number of constrained cells; over this benchmark family the dependence on the number of attributes shows no exponential growth.

#### 6.1.8 Metric

For a sampled population, index the retained marginals by *c* = 1, …, *m* and let ${\hat{\alpha }}_{c,a}$ and α_*c, a*_ denote the generated and target frequencies of cell *a* of marginal *c*. We report the mean relative constraint error


$$\begin{array}{c} \text{MRE}≜\frac{1}{m}\overset{m}{\underset{c=1}{\sum }}\underset{a}{\sum }|{\hat{\alpha }}_{c,a}-\alpha_{c,a}|, \end{array}$$ (7)

The mean relative error defined in Equation 7 is the mean over retained marginals of the L1 distance between the generated and target marginal distributions; we also report runtime. Because only marginal cells with nonzero empirical support are retained, all target frequencies are strictly positive.

### 6.2 Results on NPORS-derived scaling benchmarks

All results below use the five NPORS-derived problems described above. We vary the sampled population size *N* to study how each method behaves under increasing sampling pressure while keeping the underlying constraint system fixed.

As expected, the CP-SAT approach computes exact solutions but does not scale. In the case of 12 variables and 315 target cells, the solver proves optimality for populations up to *n* = 30 (in under 90 s). Beyond this threshold, the solver returns feasible but unproved solutions whose relative error degrades rapidly with population size. In the case of 24 variables and 3,621 target cells, the solver cannot prove optimality even for the smallest tested population (*n* = 10), and fails to find any feasible solution for *n*≥200 within the allotted time. These results are summarized in Figure 1. We emphasize that these are computational limitations of the tested formulation under the stated time budget, not proofs of mathematical infeasibility. Tractability at the population sizes used in the main experiments was not demonstrated under the tested formulation and computational budget, which is what motivates the relaxation.

The same distinction applies to classical IPF over the complete joint contingency table, which can in principle fit compatible higher-order marginal constraints exactly. We ran it wherever that table is representable: at *K* = 4 (81 cells) and *K* = 12 (354, 294 cells), full-table IPF fitted on exactly the same margins converges with a maximum margin residual below 3 × 10^−7^, and populations sampled from the fitted table reach MRE 0.011 ± 0.001 and 0.020 ± 0.001 (three seeds), comparable to or better than the released MaxEnt and raking runs on the same instances (0.027 and 0.016 at *K* = 4; 0.026 and 0.026 at *K* = 12). At *K* = 20, 28, and 40 the joint table would hold 2.3 × 10^10^, 9.1 × 10^15^, and 2.0 × 10^26^ cells respectively, so full-table IPF is excluded by representation and cost, not by an inability to handle such constraints in principle.

Figures 2, 3 summarize the relative performance of MaxEnt and Raking across problem sizes and constraint arities in 2D and 3D competence maps. Figures 4, 5 show mean relative error as a function of population size for 28- and 40-variable instances, at arity 2 and arity 3.

**Figure 2:**
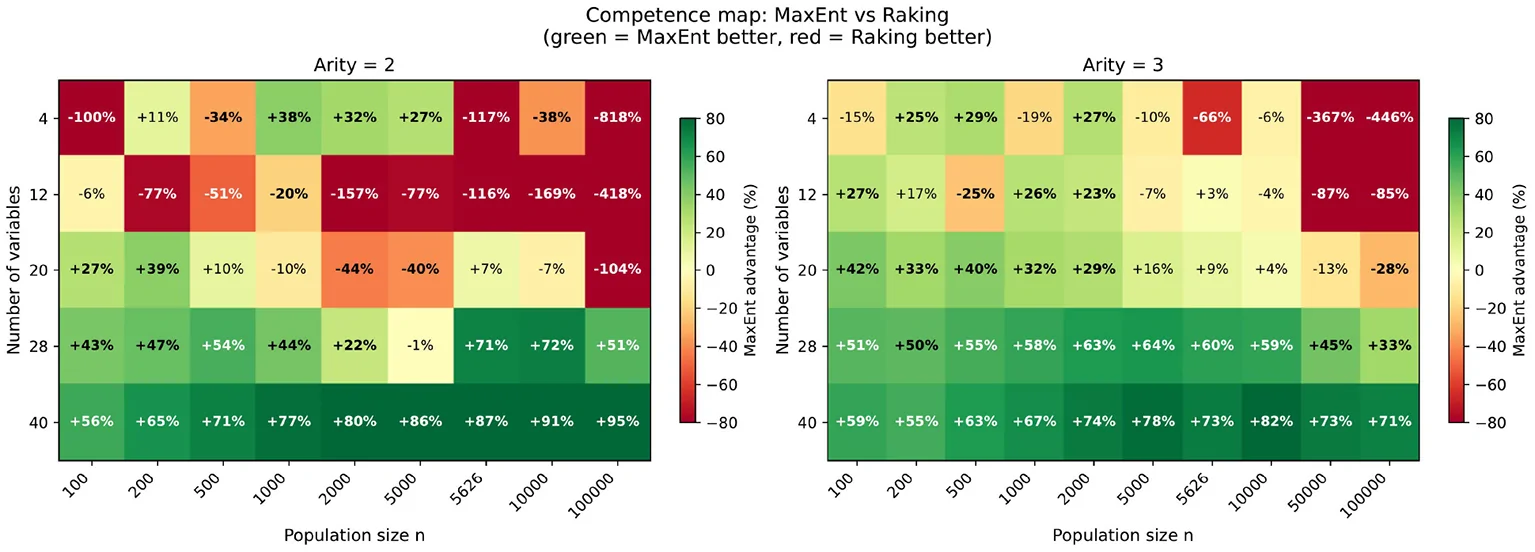
Competence map of MaxEnt vs. Raking (2D view): relative advantage as a function of population size and number of variables, for arity 2 and arity 3. Green = MaxEnt better; red = Raking better.

**Figure 3:**
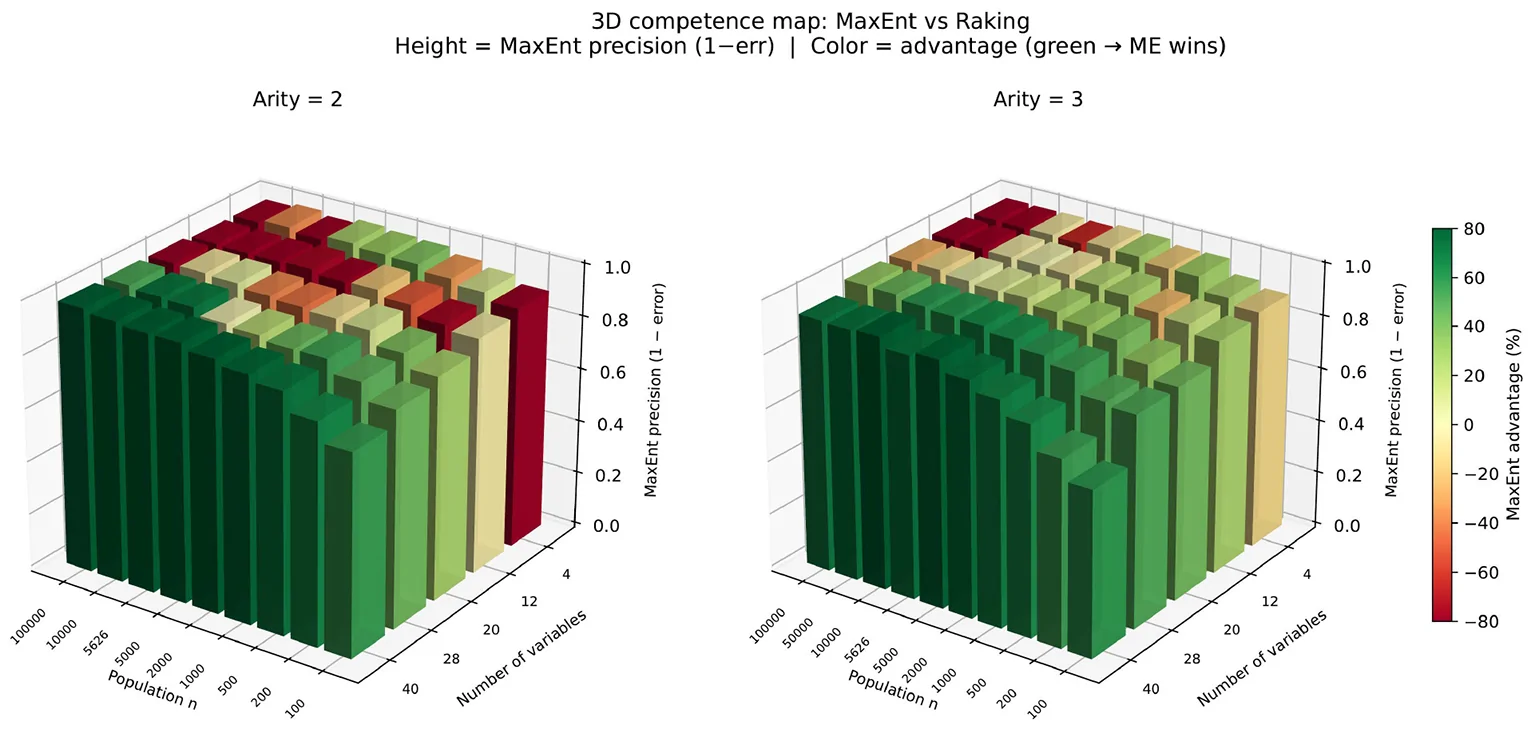
Competence map of MaxEnt vs. Raking (3D view): height encodes MaxEnt precision (1−err); color encodes relative advantage. Green regions indicate configurations where MaxEnt wins.

**Figure 4:**
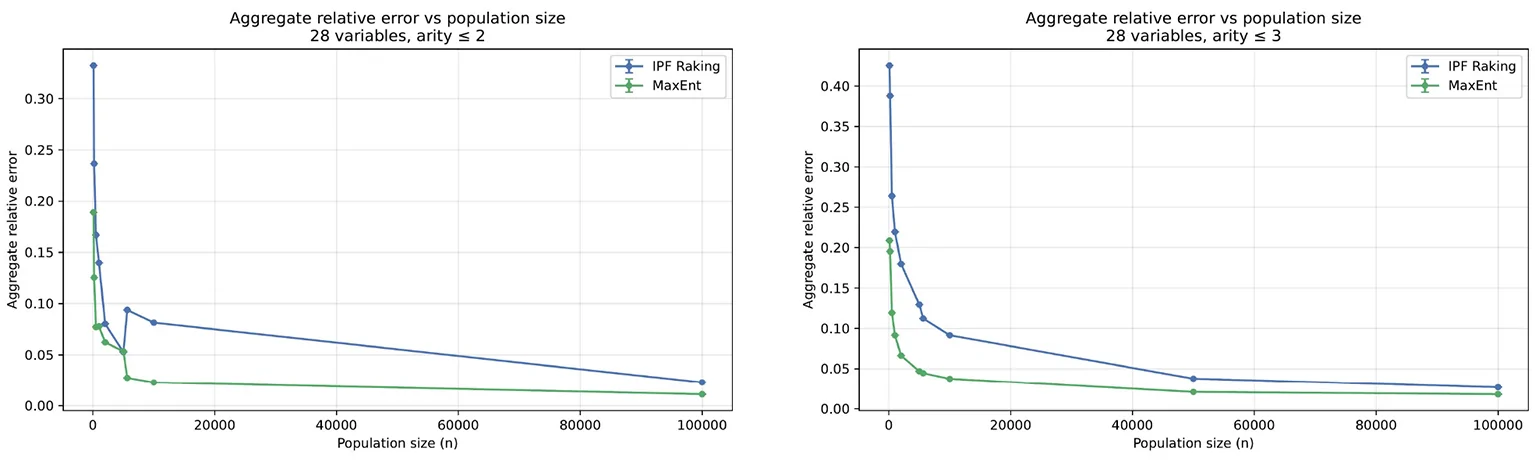
Mean relative error vs. population size for 28-variable instances (left: arity 2; right: arity 3). MaxEnt consistently outperforms Raking for small populations and high arity.

**Figure 5:**
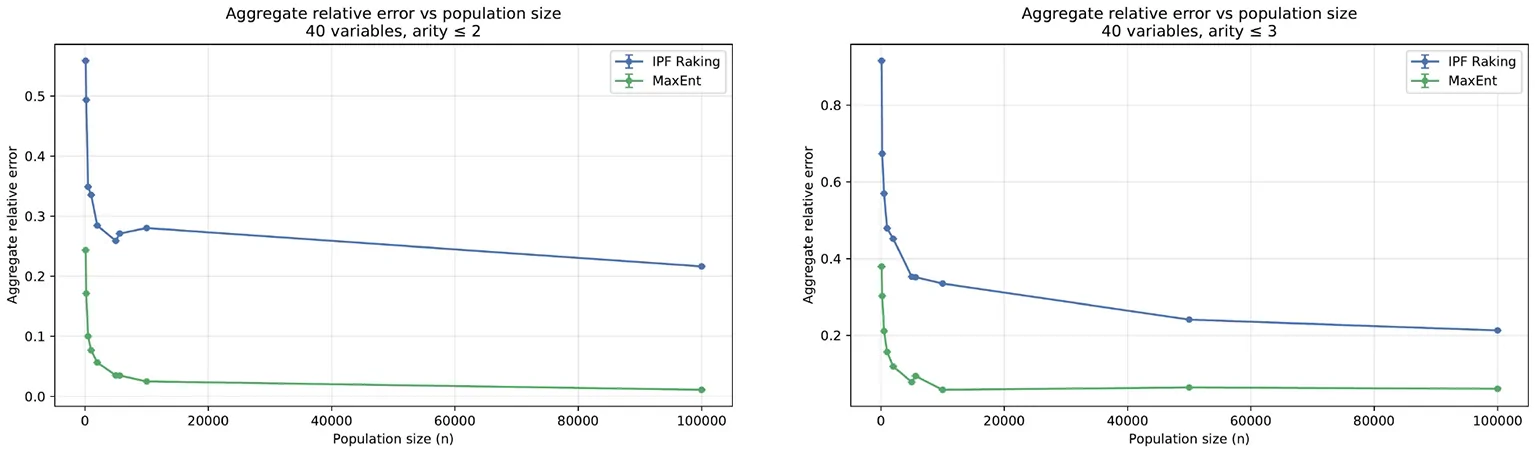
Mean relative error vs. population size for 40-variable instances (left: arity 2; right: arity 3). The advantage of MaxEnt over Raking grows with the number of variables.

### 6.3 Constraint-budget ablation: what low-order margins can and cannot recover

A natural concern is that, when only low-order margins are available, maximum entropy cannot recover information absent from the constraints, and both MaxEnt and raking are then limited by the same missing information. We test this directly on the 20-variable benchmark by fitting under three nested regimes, unary only, unary and binary, and unary, binary and ternary (all derived from the same arity-3 constraint set, so each regime is a strict subset of the next), and evaluating each fitted population on *held-out* higher-order marginals never used for fitting: a sample of 120 ternary marginals disjoint from the fitted set, and 120 four-way marginals. Table 4 reports the held-out mean relative error at *N* = 5, 626.

**Table 4:** Constraint-budget ablation on the 20-variable benchmark (*N* = 5, 626): mean relative error on held-out higher-order marginals never used for fitting, as a function of the fitting arity.

	Held-out ternary		Held-out 4-way	
Fitting constraints	MaxEnt	Raking	MaxEnt	Raking
Unary only	0.120	0.123	0.202	0.205
+ binary	0.069	0.092	0.127	0.176
+ binary + ternary	0.059	0.075	0.091	0.143

Two things are visible. With unary-only constraints the two methods are essentially indistinguishable on held-out higher-order marginals (0.120 vs 0.123 on ternary; 0.202 vs 0.205 on four-way): as expected, neither recovers structure that the constraints do not contain, and MaxEnt has no advantage from low-order information alone. The MaxEnt advantage becomes substantial once overlapping binary constraints are added and remains substantial when ternary constraints are added (held-out four-way error 0.127 vs 0.176, then 0.091 vs 0.143). This confirms the expected information-theoretic limitation, and locates the benefit of MaxEnt precisely: not in extracting higher-order information from low-order margins, but in reconciling many overlapping higher-order constraints into a single global fit that generalizes better to unseen marginals.

### 6.4 Validity of unconstrained combinations

Matching the imposed margins does not guarantee that *unconstrained* combinations are plausible. Using the arity-3 populations (*N* = 5, 626), we audit three quantities, each a fraction of generated records (the NPORS source scores 0 on all three by construction): the rate of records containing at least one attribute-value pair never observed in the source; the same rate restricted to pairs *not* in the fitted constraint set (combinations the model was never told about); and, on a held-out sample of 150 four-way marginals never used for fitting, the rate of records whose four-way combination is unobserved in the source. Table 5 reports the results for the 40-variable benchmark, where the effect is most visible.

**Table 5:** Validity audit of unconstrained combinations on the 40-variable arity-3 benchmark (*N* = 5, 626).

Method	Any never-obs. pair	Unconstrained never-obs. pair	Held-out 4-way novel
MaxEnt	0.446	0.133	0.023
Raking	0.905	0.029	0.024

Values are fractions of generated records; the source population scores 0 throughout.

Three observations follow. First, neither method eliminates unconstrained novelty: on held-out four-way marginals both produce novel combinations at similar low rates (≈2.3%). Second, overall MaxEnt is cleaner, producing far fewer never-observed pairs than raking (0.45 vs 0.90), because raking's poor constraint fit at this scale scatters mass into invalid cells. Third, and most important for this point, on the pairs it was *not* constrained on, MaxEnt's entropy-maximizing behavior introduces *more* never-observed combinations than raking (0.133 vs 0.029). Maximum entropy therefore does not, by itself, forbid structurally invalid combinations; validity is enforced only to the extent that impossibilities are encoded, either by removing impossible cells from the support (assigning them zero probability) or by adding explicit constraints with target zero. Encoding known impossibilities as hard-zero support is thus recommended in practice, and a tighter integration of structural validity into the feature set is a natural extension.

Two clarifications sharpen the reading of these rates. First, they are record-level union events: a record is counted as soon as it contains at least one never-observed pair among its many pair slots (402=780 at *K* = 40), so the rate grows mechanically with *K*. Normalized by opportunity, i.e. violating (record, pair) slots over all slots, the 0.133 unconstrained-pair rate corresponds to 3.1 × 10^−4^ of pair slots for MaxEnt vs. 4.6 × 10^−5^ for raking, and the any-pair rates become 9.3 × 10^−4^ vs. 2.2 × 10^−3^. Second, “never observed in a finite source sample” is a sampling zero, not automatically a structural impossibility; since no combination of these benchmarks is independently known to be impossible, we describe such combinations as previously unobserved rather than invalid. The synthetic family of Section 6.6, whose true support is known by construction, provides the complementary audit in which genuine structural violations are measurable.

### 6.5 Robustness to noisy and inconsistent targets

The penalized formulation in Equation 3 is motivated by targets that are noisy or mutually inconsistent. We validate this regime empirically on the 12-variable arity-3 benchmark (*N* = 5, 626), chosen because both methods perform equally well there in the noise-free case, which isolates the response to corruption from the high-dimensional effects studied above. Two corruptions are applied to the target counts. First, multiplicative noise: every cell target *t* is replaced by max(0, round(*t*(1+ε))) with $\varepsilon \sim N\left(0,\sigma^{2}\right)$ and σ∈{0.05, 0.10, 0.20}, while marginal totals are kept fixed, so the margins become mutually and globally inconsistent. Second, a deliberate contradiction: 20% of the largest unary category of the variable shared by the most higher-order margins is moved to its smallest category, while all overlapping binary and ternary margins keep the true distribution. Both methods are fitted on the corrupted targets with their standard procedures and evaluated against the *true* targets (three replicates each). Two clarifications frame the interpretation of this experiment. First, the standard MaxEnt fit of Section 5 performs stochastic gradient steps on the *hard-constraint* dual Equation 5 with a decaying learning rate; for infeasible targets that dual need not possess a finite minimizer, so the finite iterate reported here is an *early-stopped* approximation, not a solution of the penalized problem Equation 3. Table 6 should therefore be read as an empirical robustness study of the early-stopped stochastic moment-matching procedure under infeasible targets. Second, the penalized formulation itself is evaluated explicitly below, through its ridge-regularized dual Equation 6, with the fidelity parameter β and the weights *w*_*j*_ reported (Table 7).

**Table 6:** Robustness to corrupted targets on the 12-variable arity-3 benchmark (*N* = 5, 626; MRE vs true targets reported as mean ± std over three replicates).

	MRE vs true targets		MRE vs corrupted targets	
Condition	MaxEnt	Raking	MaxEnt	Raking
no corruption	0.029 ± 0.002	0.030 ± 0.001	0.029	0.030
noise σ = 0.05	0.040 ± 0.003	0.038 ± 0.006	0.058	0.058
noise σ = 0.10	0.060 ± 0.003	0.060 ± 0.008	0.100	0.103
noise σ = 0.20	0.109 ± 0.015	0.106 ± 0.014	0.193	0.195
contradiction	0.037 ± 0.001	0.026 ± 0.002	0.040	0.030

Both methods are fitted on corrupted targets; the left block evaluates the generated populations against the true (uncorrupted) targets, the right block against the corrupted targets actually fitted (means over the same replicates; stds, of similar magnitude, omitted for readability).

**Table 7:** Explicit evaluation of the penalized formulation Equation 3 via its ridge dual Equation 6 on the corrupted 12-variable benchmark (MRE vs the true targets, mean ± std over three replicates; the early-stopped column reproduces Table 6).

	Early-stopped	Penalized, *w*_*j*_ = 1			*w*_*j*_∝1/α_*j*_
Condition	(Table 6)	β = 10	β = 100	β = 1, 000	β = 100
no corruption	0.029	0.095 ± 0.002	0.035 ± 0.004	0.028 ± 0.000	0.038 ± 0.003
noise σ = 0.05	0.040	0.096 ± 0.006	0.043 ± 0.001	0.044 ± 0.003	0.047 ± 0.005
noise σ = 0.10	0.060	0.107 ± 0.014	0.065 ± 0.004	0.066 ± 0.006	0.053 ± 0.007
noise σ = 0.20	0.109	0.127 ± 0.020	0.110 ± 0.015	0.108 ± 0.014	0.094 ± 0.019
contradiction	0.037	0.100 ± 0.006	0.044 ± 0.002	0.039 ± 0.002	0.053 ± 0.002

Across all 60 penalized fits the multipliers remain bounded ($\underset{j}{\max}|\lambda_{j}|\leq 10.1$). Outside the heaviest conditions the mean fixed-point residual is 0.001 to 0.006, at the per-sweep sampling-noise scale of Table 15, consistent with convergence to the ridge-dual optimum rather than early stopping; at σ = 0.20 with β≥100 it reaches 0.005 to 0.011, so convergence there is approximate at this budget.

Three conclusions emerge. First, the early-stopped procedure degrades gracefully: neither method diverges or fails within the fitting budget when the constraint system becomes infeasible, the iterates remain bounded, and the error grows smoothly with the corruption level. Second, both methods exhibit a marked denoising property: at every noise level the generated populations are substantially closer to the true targets than to the corrupted targets they were fitted on (for instance 0.060 vs. 0.100 at σ = 0.10), because the mutually inconsistent noise cannot be satisfied jointly and the feasible compromise gravitates back toward the consistent structure. Third, at this moderate dimensionality the two methods absorb unstructured noise almost identically, and the localized contradiction is absorbed slightly better by raking (0.026 vs. 0.037), whose sequential updates let the many consistent higher-order margins override the single corrupted unary margin, whereas the global MaxEnt compromise grants the corrupted margin its weight. We present this experiment as an empirical robustness study of both fixed-budget procedures rather than as a further advantage of MaxEnt; the regime where MaxEnt dominates remains the high-dimensional overlapping one of Table 8.

**Table 8:** Mean relative constraint error (lower is better) on representative NPORS-derived scaling benchmarks.

Config	Pop. *N*	ME	Raking	Winner	Gap
12 variables					
12-var, arity=2	1,000	0.036	0.030	Raking	−17%
12-var, arity=3	100	0.129	0.176	ME	+27%
12-var, arity=3	5,626	0.026	0.026	equal	≈0
12-var, arity=3	100,000	0.017	0.009	Raking	−46%
20 variables					
20-var, arity=2	200	0.114	0.186	ME	+39%
20-var, arity=3	100	0.204	0.352	ME	+42%
20-var, arity=3	10,000	0.027	0.028	ME	+4%
20-var, arity=3	100,000	0.013	0.011	Raking	−22%
28 variables: MaxEnt wins in all configurations					
28-var, arity=2	1,000	0.078	0.140	ME	+44%
28-var, arity=3	100	0.209	0.425	ME	+51%
28-var, arity=3	10,000	0.037	0.092	ME	+59%
28-var, arity=3	100,000	0.018	0.027	ME	+33%
40 variables: largest observed gaps					
40-var, arity=2	1,000	0.077	0.336	ME	+77%
40-var, arity=3	100	0.380	0.916	ME	+59%
40-var, arity=3	5,626	0.095	0.352	ME	+73%
40-var, arity=3	100,000	0.061	0.214	ME	+71%

Gap = relative reduction over the weaker method. At *K* ≤ 12, the winner depends on arity and *N*. At *K*≥28, MaxEnt wins in all configurations we tested. MaxEnt columns report the finite-budget MCMC approximation of Section 5, not the exact MaxEnt optimum.

We now evaluate the penalized formulation Equation 3 explicitly, through its ridge-regularized dual Equation 6. In practice, optimizing Equation 6 requires no change to the MCMC sampler: relative to the hard-dual update, the gradient simply acquires the additional ridge term λ_*j*_/(β*w*_*j*_), so that


$$\lambda_{j}\leftarrow \lambda_{j}-\eta \left({\hat{𝔼}}_{p_{\lambda }}\left[f_{j}\right]-\alpha_{j}+\frac{\lambda_{j}}{\beta w_{j}}\right),$$

which makes explicit that this experiment optimizes Equation 6 directly rather than early-stopping Equation 5. We use the same corrupted 12-variable protocol as above, with *w*_*j*_ = 1 and β∈{10, 100, 1, 000}, plus a non-uniform scheme *w*_*j*_∝1/α_*j*_ (clipped to [0.2, 5] and normalized to mean one) at β = 100; three replicates per condition. Because the gradient is estimated by MCMC, convergence is assessed by the fixed-point residual $\rho_{j}=\left({\hat{\alpha }}_{j}-\alpha_{j}\right)+\lambda_{j}/\left(\beta w_{j}\right)$ averaged over the final ten iterations. Table 7 reports the results.

Three observations follow. First, the β sweep traces the expected bias-fidelity trade-off of Equation 3: at β = 10 the ridge shrinks the multipliers strongly and the error is dominated by regularization bias even without corruption (0.095 at σ = 0), while as β grows the hard-constraint limit is recovered (0.028 at β = 1, 000, matching the early-stopped 0.029). Second, at moderate and large β the finite-budget penalized fits match the early-stopped procedure within seed noise under every corruption level, so the robustness conclusions drawn from Table 6 are corroborated by direct finite-budget fits of Equation 3 itself. Third, the weights matter in the way the formulation intends: up-weighting rare cells (*w*_*j*_∝1/α_*j*_) improves accuracy under heavy unstructured noise (0.094 vs. 0.110 at σ = 0.20) at the price of slightly worse fidelity in the clean and contradiction cases.

### 6.6 Transfer to an NPORS-independent synthetic family

All benchmarks above derive from NPORS, so we test whether the observed behavior depends on that dataset's particular correlation and sparsity structure with a controlled synthetic family generated independently of it. The design is fixed and bounded: three generative regimes with constant, documented parameters, each instantiated at *K*∈{12, 28, 40}. The generative recipe of each regime is fixed; one realization of its parameters (attribute distributions, latent maps, interaction tables, forbidden pairs) is drawn once per (regime, *K*) pair with fixed seeds and then frozen. Configurations at different *K* are therefore independent realizations of the same recipe at growing size, as with the NPORS instances themselves, not nested restrictions of a single model. Attribute domain sizes cycle through {2, 3, 4, 6}. In the *independent* regime, each attribute is drawn i.i.d. from its own categorical distribution, drawn once from a Dirichlet prior and then frozen. In the *block* regime, consecutive blocks of four attributes share a latent categorical variable with six levels; each attribute in a block carries a fixed random map from the six latent levels to its own domain, and copies the mapped value of the block's latent draw with probability 0.65, otherwise drawing from its own marginal distribution, which induces within-block dependence across attributes of different domain sizes. In the *sparse higher-order* regime, an independent base is modified by two fixed mechanisms: *K*/4 disjoint ternary interaction rules, in which the third attribute of a designated triple (*a, b, c*) is overwritten by *v*_*c*_: = *f*(*v*_*a*_, *v*_*b*_) with probability 0.5 for a fixed random table *f*; and *K*/4 structural zeros, i.e. forbidden attribute-value pairs: whenever a draw contains a forbidden pair, the second attribute is redrawn from its marginal distribution restricted to the allowed values, so the support of the generative model excludes these pairs exactly. Records are i.i.d. under every regime and generated by direct sampling; no MCMC is involved in the generation, so the ground truth does not depend on the procedure under evaluation.

Three fixed samples are drawn per configuration from the generative model. A *source sample* of 10, 000 records supplies the fitted constraints: its observed marginals are the targets, with scopes selected by the same pipeline and informativeness filters as the NPORS instances and the same numbers of retained scopes per arity for the same *K*; the resulting constrained-cell counts, which depend on the support structure and are therefore not forced to match, range from 4, 442 to 11, 523 (Table 9). A *near-true reference* of 200, 000 records supplies the evaluation reference for all held-out marginals. An *independent-sample reference*, a third fresh sample of the fitted size *N* = 10, 000, is evaluated exactly like a generated population (a comparison point, not a lower bound on achievable error). Both methods are fitted at *N* = 10, 000 with their standard procedures (MaxEnt auto-tuning selects 888 to 2, 304 iterations) and 5 seeds per configuration; the seeds vary only the solvers' internal randomness (population initialization and Metropolis proposals for MaxEnt; initialization and final resampling for raking), while the generative model, the three samples, the constraint selection, and the held-out scopes are identical across seeds. The generation and evaluation code, with all fixed parameters, is in the companion repository (experiments/frontiers2026/scripts/synth_common.py and b11_synthetic.py, commit 4a0ef6d). Table 9 reports fitted-constraint MRE and held-out *L*_1_ (100 unfitted ternary and 50 unfitted binary scopes, evaluated against the near-true reference), together with the independent-sample reference.

**Table 9:** NPORS-independent synthetic family: fitted-constraint MRE (mean ± std over five seeds, population convention; as in Table 10, the 95% *t*-interval over seeds has half-width 1.39 times the reported standard deviation) and held-out *L*_1_ frequency distance against the near-true reference (means over seeds; stds are at most 0.008), with the independent-sample reference, a fresh true i.i.d. sample of the same size (a comparison point, not a lower bound).

			Fitted MRE		Held-out *L*_1_		Indep. ref.
Regime	*K*	Cells	MaxEnt	Raking	MaxEnt	Raking	(fit / held-out)
Independent	12	4,768	0.0531 ± 0.0026	0.0530 ± 0.0015	0.045	0.058	0.072 / 0.034
Independent	28	10,823	0.0531 ± 0.0009	0.0650 ± 0.0016	0.043	0.065	0.076 / 0.033
Independent	40	11,523	0.0495 ± 0.0003	0.0607 ± 0.0011	0.044	0.074	0.068 / 0.040
Block	12	4,442	0.0483 ± 0.0019	0.0564 ± 0.0030	0.047	0.086	0.062 / 0.032
Block	28	7,866	0.0447 ± 0.0013	0.1675 ± 0.0056	0.044	0.152	0.058 / 0.034
Block	40	8,263	0.0423 ± 0.0010	0.1865 ± 0.0058	0.046	0.176	0.059 / 0.040
Sparse h.o.	12	4,562	0.0539 ± 0.0010	0.0596 ± 0.0046	0.046	0.081	0.064 / 0.036
Sparse h.o.	28	9,529	0.0475 ± 0.0020	0.1254 ± 0.0022	0.041	0.116	0.060 / 0.032
Sparse h.o.	40	9,840	0.0475 ± 0.0012	0.1515 ± 0.0030	0.047	0.137	0.065 / 0.041

Held-out JS divergence follows the same pattern (MaxEnt 0.0008 to 0.0011 in every configuration; raking 0.0015 to 0.0126, growing with *K* in the dependent regimes).

Three observations follow. First, the NPORS pattern transfers: in both dependence regimes MaxEnt's error is essentially flat in *K* (fitted 0.042 to 0.054; held-out at 1.1 to 1.5 times the independent-sample reference in all nine configurations) while raking degrades sharply as *K* grows (fitted 0.056 to 0.187 in the block regime, 0.060 to 0.152 in the sparse higher-order regime), so the advantage observed on NPORS does not depend on NPORS's particular correlation or sparsity structure. Because each *K* is a separate realization of the recipe, the cross-*K* trend mixes dimensionality with realization effects; the method-to-method comparison at each configuration, which is the object of this test, is unaffected, since both methods fit the same realized instance. Second, the independent regime acts as a control: with no dependence to reconcile, the gap narrows markedly without disappearing (fitted 0.053 vs. 0.053 at *K* = 12; held-out 0.044 vs. 0.074 at *K* = 40, against 0.046 vs. 0.176 in the block regime), which is consistent with, though does not by itself establish, the structural explanation of Section 7: the MaxEnt advantage grows with the amount of overlapping structure to reconcile. Third, the structural-zero condition makes true validity measurable (neither method receives any structural information beyond the margins): at *K* = 12, where raking fits well, MaxEnt produces *more* true structural violations (2.5 of records vs. 0.6%), confirming the concern of Section 6.4 on a case with known ground truth; at *K* = 28 and *K* = 40 the ranking reverses dramatically (3.3 vs. 11.6%, then 5.2 vs. 34.9%) because raking's degraded fit scatters mass into forbidden pairs. Encoding known impossibilities as zero-probability support therefore remains the recommended remedy for both methods. These results demonstrate transfer to the tested synthetic regimes; they do not establish broad real-world generalizability.

## 7 Discussion

### 7.1 MaxEnt as a global optimizer

From an algorithmic standpoint, the proposed approach can be interpreted as an approximate solver for Sum-based multi-way cardinality CSPs. Exact CP or MIP formulations search directly for integer-feasible contingency tables. In contrast, the maximum-entropy relaxation optimizes a single global objective over the multiplier vector λ and returns a distribution over complete assignments. The learned exponential-family model therefore plays the role of a relaxed solver: it identifies regions of the solution space that best satisfy the constraints in aggregate and supports principled sampling rather than arbitrary solution selection.

Crucially, MaxEnt performs a *global* update of all Lagrange multipliers λ, finding one trade-off across all constraints at once. Raking, by contrast, applies sequential one-constraint-at-a-time rescalings, so improvements on one marginal can partially undo earlier corrections on overlapping marginals. On the NPORS-derived benchmarks, this interference becomes more pronounced as *K* and the constraint arity increase, which helps explain the larger residual errors observed for raking in the high-dimensional settings.

### 7.2 Empirical comparison: MaxEnt vs. raking

On the NPORS-derived scaling benchmarks, the most salient factor governing relative performance is *the number of variables* *K*, not the sampled population size *N* alone. For *K* ≤ 12, raking remains a strong baseline and can win at arity 2 or at large *N* even under arity 3. Around *K* = 20, the picture becomes mixed: MaxEnt is more reliable at arity 3, whereas neither method dominates uniformly at lower arity. For *K*≥28, MaxEnt outperforms raking in all tested configurations, with the largest gaps appearing on the 40-variable problems.

A plausible structural explanation is that the number of overlapping constraints grows much faster than *N* as *K* increases, increasing interference between sequential raking updates while leaving the MaxEnt fit governed by a single global objective. We present this as an empirical interpretation of the observed results rather than as a general impossibility claim.

A structural limitation deserves emphasis: maximum entropy cannot recover information that is absent from the constraints. Given only unary, or only unary and binary, margins, the maximum-entropy solution is the least-committal distribution consistent with them and introduces no higher-order structure; it should not be read as discovering rare high-order combinations unless these are represented in the feature set, the constraints, or an external prior. In the unary-only regime MaxEnt and raking fit essentially the same information, and we neither observe nor claim a substantial advantage; once overlapping binary margins are added, a substantial held-out advantage appears (Section 6.3), reflecting the reconciliation of overlapping constraints rather than the recovery of absent information. The advantage we report grows with the number of variables and the density of overlapping higher-order constraints, that is, with the difficulty of reconciling many constraints at once, not with lower information content.

Table 8 reports mean relative constraint error for representative configurations spanning five problem sizes and both arities.

To quantify sampling variability, every configuration of Table 8 was re-run with five independent seeds (three seeds for *N* = 100, 000, whose MCMC cost is proportional to *N*; the four *N* = 100, 000 configurations were subsequently extended to five seeds as well, so every configuration now rests on five seeds). Table 10 reports the mean and standard deviation of the MRE over seeds, together with the mean absolute *L*_1_ error per marginal (in individuals), which complements the relative view. Seed variability is small relative to the gaps between MaxEnt and raking wherever we claim an advantage: the largest standard deviation in the table (0.034, raking at *K* = 40, *N* = 100) is more than an order of magnitude below the corresponding gap, and in the high-dimensional regime the intervals are widely separated (for instance 0.072 ± 0.002 vs. 0.131 ± 0.011 at *K* = 28, arity 2, *N* = 1, 000). The multi-seed means are close to the single-seed values of Table 8 except in the smallest-population configurations, where a single draw is noisiest: deviations reach 0.070 in absolute MRE (raking at *K* = 40, *N* = 100) and 0.048 at *K* = 28, *N* = 100, while for *N*≥1, 000 they remain below 0.01. Outside one borderline case discussed next, the winner is unchanged in every configuration. Multi-seed averaging refines one borderline case: at *K* = 12, arity 3, *N* = 100, the apparent single-seed MaxEnt advantage (0.129 vs. 0.176) is not robust, the multi-seed intervals overlapping (0.150 ± 0.019 vs. 0.150 ± 0.011); the empirical guidance for small problems should therefore read “no reliable difference” rather than a MaxEnt advantage at *K* ≤ 12. All conclusions for *K*≥20, where our claims lie, are unchanged and strengthened by the tight intervals.

**Table 10:** Multi-seed variability on the configurations of Table 8: MRE mean ± standard deviation over five seeds, now for all configurations (the four *N* = 100, 000 rows were extended from three to five seeds in this revision, and the MRE columns are restated at four decimals, in magenta), and mean absolute *L*_1_ error per marginal in individuals.

		MRE (mean ±std)		Abs. *L*_1_ per marginal	
Config	Pop. *N*	MaxEnt	Raking	MaxEnt	Raking
12-var, arity=2	1,000	0.0347 ± 0.0043	0.0309 ± 0.0048	35	31
12-var, arity=3	10	0.1505 ± 0.0186	0.1501 ± 0.0107	15	15
12-var, arity=3	5,626	0.0283 ± 0.0023	0.0287 ± 0.0021	159	161
12-var, arity=3	100,000	0.0161 ± 0.0003	0.0084 ± 0.0007	1,617	833
20-var, arity=2	200	0.0922 ± 0.0101	0.1536 ± 0.0166	18	31
20-var, arity=3	100	0.2027 ± 0.0136	0.3400 ± 0.0288	20	34
20-var, arity=3	10,000	0.0271 ± 0.0017	0.0293 ± 0.0016	272	293
20-var, arity=3	100,000	0.0132 ± 0.0008	0.0106 ± 0.0004	1,326	1,060
28-var, arity=2	1,000	0.0724 ± 0.0017	0.1314 ± 0.0109	72	131
28-var, arity=3	100	0.2237 ± 0.0162	0.3771 ± 0.0200	22	38
28-var, arity=3	10,000	0.0339 ± 0.0010	0.0922 ± 0.0037	339	922
28-var, arity=3	100,000	0.0179 ± 0.0003	0.0264 ± 0.0005	1,790	2,641
40-var, arity=2	1,000	0.0768 ± 0.0019	0.3267 ± 0.0186	77	327
40-var, arity=3	100	0.3815 ± 0.0111	0.8464 ± 0.0335	38	85
40-var, arity=3	5,626	0.0956 ± 0.0017	0.3573 ± 0.0106	538	2,010
40-var, arity=3	100,000	0.0616 ± 0.0005	0.2206 ± 0.0057	6,160	22,058

Standard deviations use the population convention (denominator *n*); with five seeds throughout, the 95% *t*-interval over seeds therefore has half-width 1.39 times the reported standard deviation in every row. Seed-to-seed variability quantifies run randomness of the finite-budget procedures, not uncertainty about generalization across datasets. Extending the four *N* = 100, 000 configurations from three to five seeds changes no winner; across the full table the high-dimensional conclusions are unchanged, while the small-population 12-variable arity-3 case shows no reliable difference (see text).

Because no single constraint-fit statistic captures every aspect of synthetic-population quality, Table 11 complements the *L*_1_-based errors with a bounded distributional measure: the Jensen-Shannon divergence (base 2, in [0, 1]) between the generated populations and the source on held-out marginals never used for fitting (50 unfitted binary and 100 unfitted ternary scopes per instance). The ranking agrees with the *L*_1_-based conclusions: the two methods are equivalent at *K* = 12 and the MaxEnt advantage grows with *K*. The evaluation reported in this paper thus covers fitted-constraint accuracy (Tables 8, 10), generalization to held-out marginals in *L*_1_ (Table 4) and in JS divergence (Table 11), audits of previously unobserved combinations (Table 5), robustness to corrupted targets (Tables 6, 7), and runtime and memory (Table 12); dimensions beyond these, such as downstream-task utility or disclosure risk, are outside the paper's claims.

**Table 11:** Held-out distributional quality on the arity-3 benchmarks (*N* = 5, 626, released populations): mean Jensen-Shannon divergence (base 2) and mean *L*_1_ frequency distance over 150 held-out marginal scopes (50 unfitted binary, 100 unfitted ternary) between generated populations and the NPORS source.

	Held-out JS divergence		Held-out *L*_1_ distance	
Problem	MaxEnt	Raking	MaxEnt	Raking
12-Var	0.0025	0.0021	0.028	0.030
20-Var	0.0025	0.0056	0.050	0.075
28-Var	0.0044	0.0133	0.070	0.133
40-Var	0.0138	0.0463	0.138	0.301

**Table 12:** Measured end-to-end fitting cost (wall-clock seconds, mean ± std over three seeds) and peak resident memory on the arity-3 benchmarks (*N* = 5, 626).

	MaxEnt		Raking	
Problem	Time (s)	Peak mem. (MB)	Time (s)	Peak mem. (MB)
4-Var	5.4 ± 0.2	73	1.9 ± 0.0	66
12-Var	78.7 ± 1.8	91	14.1 ± 0.1	74
20-Var	325.1 ± 10.2	96	33.2 ± 0.2	80
28-Var	544.2 ± 11.8	99	54.6 ± 1.2	80
40-Var	85.8 ± 1.2	111	251.7 ± 0.5	90

MaxEnt uses the per-instance iteration budgets of Table 3; raking uses tolerance 10^−6^ with a 500-iteration cap.

### 7.3 Empirical guidance

The empirical results above support a benchmark-specific rule of thumb, summarized in Table 13.

**Table 13:** Empirical guidance on the NPORS-derived scaling benchmarks.

Situation	Guidance
*K* ≤ 12, arity = 2	Raking is a strong baseline and can be preferred when speed is the main concern.
*K* ≤ 12, arity = 3, small or moderate *N*	No reliable difference across seeds (Table 10); raking preferable on speed grounds.
*K*≈20	Method choice becomes regime-dependent; MaxEnt is safer when ternary interactions matter.
*K*≥28	MaxEnt consistently outperformed raking on our benchmarks.
*K* = 40	The largest empirical gaps favor MaxEnt by a wide margin.

These recommendations summarize observed trends and should not be read as universal guarantees.

The *K* ≤ 12, arity-3 entry already incorporates the multi-seed analysis of Table 10: the advantage observed in single runs falls within seed variability, so the two methods should be considered equivalent there. The recommendations for *K*≥20 are unchanged.

### 7.4 Scalability and practical limits

Computational cost is governed by the number of retained constrained cells, the population size *N*, the optimization budget, and the sampler's mixing behavior. This section therefore treats the constrained-cell count and the wall-clock budget as the primary scaling variables, with the attribute count *K* serving as an instance label for the benchmark family. Because fitting uses MCMC over a working population rather than enumeration of the joint space X (which is astronomically large: about 9 × 10^15^ states for the 28-attribute benchmark and 2 × 10^26^ for the 40-attribute one), the cost empirically scales without exponential growth in the tested benchmark family. The runtime is governed by the population size *N*, the number of constrained cells (features), the number of MCMC sweeps per update, and the mixing behavior of the sampler; the number of constrained cells can itself grow rapidly with attribute domain sizes, interaction order, and the number of retained interactions, so no polynomial-in-*K* guarantee is claimed for the general problem class. Table 3 reports, for the arity-3 benchmarks, the number of constrained cells, the iteration budget, and the measured wall-clock time per outer iteration (ten MCMC sweeps each). The per-iteration cost grows no faster than linearly (in fact sublinearly at the largest sizes) with the number of constrained cells, from 0.09 s at 158 cells to 1.34 s at 19, 409 cells, and never exponentially with *K*; the number of parameters equals the number of constrained cells (Table 2), not |X|.

Beyond the per-iteration cost, Table 12 reports measured end-to-end wall-clock time and peak resident memory for both methods on the arity-3 benchmarks at *N* = 5, 626 (three seeds; each fit runs in a dedicated process so that its peak memory is measured in isolation). The end-to-end times are lower than the product of Table 3's per-iteration figures and iteration counts because those figures were measured on short probes that amortize fixed setup costs over few iterations; the steady-state per-iteration cost is correspondingly lower (for instance 0.59 s rather than 1.11 s at *K* = 28), while the scaling behavior with the number of constrained cells is the same. Two observations follow. First, raking is substantially faster per fit for *K* ≤ 28 (for instance 55 vs. 544 s at *K* = 28); the accuracy gap documented in Table 8 is therefore bought at a real but bounded computational premium. Second, the ordering reverses at *K* = 40, where MaxEnt completes in 86s against 252 for raking: MaxEnt's cost is driven by the per-instance iteration budget (120 outer iterations there), while raking's cost grows with the ever larger number of overlapping margins it must process at each of its up to 500 iterations. We stress that this reversal is a consequence of the instance-specific optimization budget, not evidence that computational complexity improves with dimensionality: at matched iteration budgets the 40-variable per-iteration cost is the largest of the family (Table 3). To substantiate this, we refit the 40-variable instance with the 28-variable budget of 926 iterations (three seeds): the error improves from 0.096 ± 0.002 to 0.084 ± 0.001 at a wall-clock cost of 554 s, the largest of the family. The 120-iteration budget is therefore a deliberate cost-accuracy trade-off rather than a sufficient budget, and the fast 40-variable runtime in Table 12 reflects that smaller budget, not an intrinsically easier problem. Peak memory is modest for both methods (below 1, 15 MB in all configurations) and is dominated by the *N*×*K* working population and the constrained-cell tables.

To compare the two methods under consistent computational budgets, Table 14 reports accuracy vs. wall-clock time as ladders of complete runs at increasing iteration budgets, for both methods, on the same instances (*K* = 28 and 40, arity 3, *N* = 5, 626), the same machine, three seeds per rung. Timing covers the full solve call: initialization, fitting, and generation of the final population; evaluation is excluded. MaxEnt rungs share one learning-rate schedule per instance, tuned for the largest budget, so shorter rungs are true truncations of the same trajectory; raking runs with its convergence test disabled so the iteration count is exact.

**Table 14:** Accuracy vs. wall-clock time under consistent budgets (arity-3 benchmarks, *N* = 5, 626, mean ± std over three seeds; complete runs, one machine; timing = initialization + fit + final population generation).

	28-Var		40-Var	
MaxEnt budget (outer iterations)	wall (s)	MRE	wall (s)	MRE
50	44	0.070 ± 0.003	51	0.127 ± 0.001
100 (120 at 40-Var)	78	0.052 ± 0.001	100	0.103 ± 0.005
200	143	0.047 ± 0.001	154	0.093 ± 0.001
400	274	0.044 ± 0.002	282	0.088 ± 0.000
926	620	0.044 ± 0.001	616	0.084 ± 0.001
120, released schedule	–	–	98	0.096 ± 0.002
Raking, 1 to 500 iterations	0.2 – 53	0.116 – 0.124	0.6 – 242	0.353 – 0.362

MaxEnt rungs truncate one shared schedule per instance (lr decay tuned for 926 iterations); the extra 40-Var row uses the released 120-iteration schedule of described in Section 5.1. Raking rows span its whole ladder: its accuracy is flat from the second iteration onward, so a single range summarizes all rungs.

Two conclusions follow. First, raking's accuracy is essentially budget-insensitive: it reaches its plateau within one or two iterations (under a second at *K* = 28) and additional budget does not improve it, consistent with the oscillation analysis above. MaxEnt outperforms raking at every reported MaxEnt runtime (0.070 vs. 0.120 at *K* = 28 from 44 s; 0.127 vs. 0.354 at *K* = 40 from 51 s) and improves monotonically with time, the observed error plateauing by about 400 iterations at *K* = 28 (0.044, unchanged at 926) while still improving at 926 iterations at *K* = 40 (0.084), in agreement with the matched-budget refit above. Shorter budgets were not evaluated, and we make no claim about that regime. Second, schedules matter at small budgets: at the same 120-iteration budget on the 40-variable instance, the released decay reaches 0.096 vs. 0.103 for the truncated long-horizon schedule, so the per-instance tuning is part of the reported cost-accuracy trade-off.

On the raking side, the comparison does not depend on a runtime budget. We re-ran raking with its convergence tolerance disabled for up to 2, 000 iterations and recorded the error at every iteration. The weighted *L*_1_ error plateaus by about 50 iterations and is then essentially constant: for the 28-variable benchmark it is 82, 770 at iteration 10 and about 83, 150 from iteration 100 through 2, 000, and the sampled error is unchanged (mean relative error 0.112 at 500 iterations vs. 0.124 at 2, 000), while MaxEnt reaches 0.044. The 40-variable benchmark behaves identically: raking's error is unmoved by a fourfold increase in iterations (0.352 at 500 vs. 0.368 at 2, 000), against MaxEnt's 0.095. The residual gap is therefore not an artifact of an early stop or a fixed runtime; it reflects oscillation between overlapping, mutually inconsistent margins that additional iterations do not resolve. Finally, we state the practical limits of the present implementation: the binding costs are the number of retained constrained cells and MCMC mixing time as arity and *K* grow, together with the accuracy floor set by MCMC sampling noise at small *N*. Scaling substantially further would call for better proposals, tempering, or a compiled or factorized sampler.

Because the optimization gradient is estimated from finite MCMC runs, all reported results should be read as the empirical performance of a fixed-budget stochastic procedure rather than as evidence of reliable convergence to the exact MaxEnt optimum. To substantiate the stability of the reported moment estimates in the two largest cases, Table 15 reports compact sampler diagnostics for the 28- and 40-variable arity-3 benchmarks: the Metropolis acceptance rate at the beginning, middle, and end of the fit; the per-cell coefficient of variation of the estimated constraint frequencies over 200 continued sweeps at frozen multipliers (statistics over the final 100 sweeps to discard the transient), with the effective sample size of the total-*L*_1_-error series; and the sensitivity of the final error to the number of sweeps per update and to the post-fit sampling budget.

**Table 15:** Compact MCMC diagnostics for the 28- and 40-variable arity-3 benchmarks (*N* = 5, 626, default fitting parameters, one instrumented fit each).

Diagnostic	28-Var	40-Var
Total sweeps during the fit	9, 310	1, 250
Acceptance rate (first / middle / last 10 sweeps)	1.00/0.25/0.25	1.00/0.29/0.26
Final MRE of the instrumented fit	0.045	0.094
Frozen multipliers: 200 continued sweeps, statistics over the final 100		
CV of per-cell frequency estimates (mean / max over cells)	0.41/9.9	0.67/9.9
Relative std of the total-*L*_1_-error series	5.4%	3.0%
ESS of the total-*L*_1_-error series (out of 100)	9.3	4.6
Final MRE vs number of MCMC sweeps per update (three seeds each; default 10)		
5 sweeps per update	0.0433 ± 0.0002	0.1014 ± 0.0069
10 sweeps per update	0.0436 ± 0.0013	0.0958 ± 0.0021
20 sweeps per update	0.0443 ± 0.0033	0.0981 ± 0.0007
MRE of fresh populations sampled from the fitted multipliers (five draws each)		
burn-in 25 sweeps	0.0480 ± 0.0019	0.1824 ± 0.0029
burn-in 50 sweeps	0.0438 ± 0.0022	0.1277 ± 0.0031
burn-in 100 sweeps	0.0436 ± 0.0022	0.1030 ± 0.0026

Top block: Metropolis acceptance rate during the fit (the initial value 1.00 reflects the uniform initialization λ = 0). Second block: sampler variability at frozen multipliers, i.e. 200 further sweeps at the fitted λ with no updates: per-cell coefficient of variation of the single-sweep constraint-frequency estimates (the maximum is attained by near-empty cells occupied in a single sweep), and relative standard deviation and effective sample size (ESS) of the total-*L*_1_-error series. Bottom blocks: sensitivity of the final error to the per-update sweep budget (refits, three seeds per setting) and to the burn-in used when sampling fresh populations from the same fitted multipliers (five draws per setting).

Three observations follow. First, after the transient from the uniform initialization the acceptance rate stabilizes at a moderate level, 0.25 at *K* = 28 throughout the fit and 0.29 to 0.26 over the second half at *K* = 40, still drifting slowly downward at the end, consistent with the deliberately limited 120-iteration budget discussed above; neither case shows sampler degeneracy. Second, at frozen multipliers the single-sweep estimates of individual cell frequencies are noisy (mean CV 0.41 and 0.67, dominated by rare cells; for typical constrained-cell frequencies this is an absolute per-sweep standard deviation of order 10^−3^, the sampling-noise scale against which the fixed-point residuals of Table 7 are read), and the total-*L*_1_-error series is strongly autocorrelated (ESS 9.3 and 4.6 out of 100), yet its relative dispersion is only 5.4% and 3.0%: the aggregate constraint mismatch that drives the reported errors is stable even though individual rare-cell estimates fluctuate. Third, the final error is insensitive to halving or doubling the per-update sweep budget at both sizes (all settings within seed noise of the default), while sampling a fresh population from the fitted multipliers requires a burn-in that grows with *K*: 50 sweeps suffice at 28 variables, whereas at 40 variables the error still improves from 0.182 to 0.103 between 25 and 100 sweeps, approaching but not matching the 0.094 obtained by the fit-time population, whose effective burn-in is the full 1, 250-sweep trajectory. The diagnostics therefore support the stability of the reported fixed-budget results while quantifying the slower mixing at *K* = 40 already noted among the practical limits above.

To probe this stability at greater depth, Table 16 extends the frozen-multiplier diagnostic with four independently initialized chains of 2, 000 sweeps each (ten times the previous budget), with statistics over the final 1, 000 sweeps of each chain and cells stratified by target frequency so that rare cells are assessed separately. We stress the framing: this is a diagnostic of sampling from the fitted distribution *p*_λ_; it quantifies how reliably the sampler explores that distribution and does not certify that the fitting phase reached the exact MaxEnt optimum.

**Table 16:** Long-chain sampling diagnostics at frozen multipliers for the 28- and 40-variable arity-3 benchmarks (*N* = 5, 626): four independently initialized chains of 2, 000 sweeps at the fitted λ (statistics over the final 1, 000 sweeps of each chain).

Diagnostic	28-Var	40-Var
Aggregate total-*L*_1_ series (per chain, final 1, 000 sweeps)		
Mean over chains	30, 720	74, 693
ESS per chain (min-max, out of 1, 000)	173 – 294	34 – 65
Relative Monte Carlo standard error	0.28% – 0.37%	0.26% – 0.40%
Split-$\hat{R}$ across the four chains	1.001	1.027
All constrained cells by target frequency: mean CV / mean |estimate−target|/ mean occupancy		
< 0.001 (1, 452 and 10, 513 cells)	0.96/0.0003/0.77	1.02/0.0002/0.70
0.001 – 0.01 (1, 541 and 7, 096 cells)	0.28/0.0005/0.99	0.31/0.0006/0.99
0.01 – 0.1 (1, 374 and 1, 563 cells)	0.08/0.0004/1.00	0.09/0.0011/1.00
≥0.1 (267 and 237 cells)	0.02/0.0009/1.00	0.02/0.0043/1.00
12 selected cells (three per stratum): split-$\hat{R}$ range	0.999 – 1.006	0.999 – 1.003

CV and occupancy are per-cell statistics of the single-sweep frequency estimates, averaged within each target-frequency stratum and over chains; occupancy is the fraction of sweeps in which the cell is nonempty. Split-$\hat{R}$ follows the standard split-chain construction; no selected cell had a constant trace at this trace length (constant traces would be excluded from $\hat{R}$ and reported through occupancy).

Three observations follow. First, chain agreement is good at the cell level at both sizes: split-$\hat{R}$ lies between 0.999 and 1.006 over the selected cells, no cell trace is constant over 2, 000 sweeps, and the aggregate mismatch is estimated with a relative Monte Carlo standard error below 0.4% everywhere. Second, the diagnostics quantify the slower mixing at *K* = 40 rather than dispose of it: the aggregate series carries about five times fewer effective samples per chain (34 to 65 vs. 173 to 294 per 1, 000 sweeps) and its split-$\hat{R}$ of 1.027 exceeds the customary 1.01 threshold, so aggregate-level estimates at *K* = 40 should be read with that reservation. Third, rare cells are individually noisy per sweep (CV near 1, occupied in 70 to 77% of sweeps below frequency 0.001) yet their chain-averaged estimates sit within a few 10^−4^ of their targets, while the largest absolute deviations appear in the most common cells at *K* = 40 (mean 0.004): the latter reflect the budget-limited fit of the multipliers, not sampling error, consistent with the fixed-budget framing above.

## 8 Conclusion

We presented a maximum-entropy relaxation of multi-way cardinality-constrained population synthesis. By interpreting conjunction-defined cardinality requirements as expectation constraints, we obtain an exponential-family model whose parameters can be learned by convex optimization. On the NPORS-derived scaling benchmarks, MaxEnt is especially attractive when the number of attributes and the density of higher-order interactions increase, whereas generalized raking remains competitive on smaller, lower-arity problems.

Two directions follow naturally from this study. First, the penalized formulation Equation 3 is now evaluated explicitly on one corrupted benchmark through its ridge-regularized dual Equation 6, with the fidelity weight β and the weights *w*_*j*_ reported (Section 6.5); extending this evaluation systematically to larger problems and to genuinely noisy real-world targets remains future work. Second, exact or near-exact repair procedures could be layered on top of MaxEnt samples when selected marginals must be matched exactly. Extending the same evaluation to higher arities and approximate expectation estimation is also an important next step.

## Data Availability

Publicly available datasets were analyzed in this study. This data can be found here: https://www.pewresearch.org/methods/dataset/2024-national-public-opinion-reference-survey-npors/.

## Footnotes

1 For example, Karpathy describes using LLM-generated synthetic conversations to inject identity and behavior into a small language model (Karpathy, 2025).

2 BORN indicates whether the respondent identifies as a born-again or evangelical Christian, DEVICE1A indicates cell phone ownership, and EMINUSE indicates whether the respondent uses the internet or email.

## References

[B1] Beckman R. J. Baggerly K. A. McKay M. D. (1996). Creating synthetic baseline populations. Transp. Res. Part A 30, 415–429. doi: 10.1016/0965-8564(96)00004-3

[B2] Bishop Y. M. M. (1967). Multidimensional contingency tables: cell estimates . J. Am. Stat. Assoc. 62, 567–576.

[B3] Borisov V. Seßler K. Leemann T. Pawelczyk M. Kasneci G. (2023). “*Language models are realistic tabular data generators*,” in *The eleventh international conference on learning representations* (Kigali: ICLR).

[B4] Borysov S. S. Rich J. Pereira F. C . (2019). How to generate micro-agents? A deep generative modeling approach to population synthesis. Transp. Res. Part C: Emerg. Technol. 106, 73–97. doi: 10.1016/j.trc.2019.07.006

[B5] Chambers R. Skinner C. (2003). Anal. Survey Data. (Chichester: Wiley).

[B6] Cooper G. F. (1990). The computational complexity of probabilistic inference using bayesian belief networks. Artificial Intelligence 42, 393–405. doi: 10.1016/0004-3702(90)90060-D

[B7] Csiszár I. (1975). I-divergence geometry of probability distributions and minimization problems. Annals Probabil. 3, 146–158. doi: 10.1214/aop/1176996454

[B8] Darroch J. N. Ratcliff D. (1972). Generalized iterative scaling for log-linear models. Annals Mathemat. Stat. 43, 1470–1480. doi: 10.1214/aoms/1177692379

[B9] Deming W. E. Stephan F. F. (1940). On a least squares adjustment of a sampled frequency table when the expected marginal totals are known. Annals Mathemat. Stat. 11, 427–444. doi: 10.1214/aoms/1177731829

[B10] Deville J.-C. Särndal C.-E. (1992). Calibration estimators in survey sampling. J. Am. Stat. Assoc. 87, 376–382. doi: 10.1080/01621459.1992.10475217

[B11] Fienberg S. E. (1980). The Analysis of Cross-Classified Categorical Data. Cambridge, MA: MIT Press.

[B12] Garrido S. Borysov S. S. Pereira F. C. Rich J. (2020). Prediction of rare feature combinations in population synthesis: application of deep generative modelling. Transp. Res. Part C: Emerg. Technol. 120:102787. doi: 10.1016/j.trc.2020.102787

[B13] Jaynes E. T. (1957). Information theory and statistical mechanics. Phy. Rev. 106:620–630. doi: 10.1103/PhysRev.106.620

[B14] Karpathy A. (2025). Guide: infusing identity to your nanochat. Available online at: https://github.com/karpathy/nanochat/discussions/139 (Accessed March 18, 2026).

[B15] Kotelnikov A. Baranchuk D. Rubachev I. Babenko A. (2023). “TabDDPM: modelling tabular data with diffusion models,” in Proceedings of the 40th international conference on machine learning (ICML), (Honolulu, HI: PMLR), 17564–17579.

[B16] Lim S. Y. Yun H. Bansal P. Kim D.-K. Kim E.-J. (2025). A large language model for feasible and diverse population synthesis. Accepted, Transp. Res. Part C. 185:105581. doi: 10.1016/j.trc.2026.105581

[B17] Lomax N. Norman P. (2016). Estimating population attribute values in a table: “get me started in” iterative proportional fitting. Professional Geo. 68, 451–461. doi: 10.1080/00330124.2015.1099449

[B18] Paglieri D. Cross L. Cunningham W. A. Leibo J. Z. Vezhnevets A. S. (2026). Persona generators: generating diverse synthetic personas at scale. arXiv:2602.03545v1.

[B19] Pearl J. (1988). Probabilistic Reasoning in Intelligent Systems: Networks of Plausible Inference. Burlington, MA: Morgan Kaufmann. doi: 10.1016/B978-0-08-051489-5.50008-4

[B20] Petit T. Pachot A. (2025). “Exact synthetic populations for scalable societal and market modeling,” in Integration of constraint programming, artificial intelligence, and operations research: 23rd international conference (Rabat, CPAIOR), 404. doi: 10.1007/978-3-032-27242-3_24

[B21] Pew Research Center (2024). 2024 National Public Opinion Reference Survey (NPORS). Available online at: https://www.pewresearch.org/methods/dataset/2024-national-public-opinion-reference-survey-npors/ (Accessed March 27, 2026).

[B22] Régin J.-C. (1996). “Generalized arc consistency for global cardinality constraint,” in AAAI/IAAI, Vol. 1 (Portland), 209–215.

[B23] Rossi F. van Beek P. Walsh T. (2006). Handbook of Constraint Programming, volume 2 of Foundations of Artificial Intelligence. Amsterdam: Elsevier.

[B24] Sakellariou J. Tria F. Loreto V. Pachet F. (2017). Maximum entropy models capture melodic styles. Sci. Rep. 7:9172. doi: 10.1038/s41598-017-08028-4 28835642 PMC5569059

[B25] Solatorio A. V. Dupriez O. (2023). REaLTabFormer: generating realistic relational and tabular data using transformers. arXiv:2302.02041.

[B26] Sun L. Erath A. (2015). A Bayesian network approach for population synthesis. Transport. Res. Part C: Emerg. Technol. 61, 49–62. doi: 10.1016/j.trc.2015.10.010

[B27] Voas D. Williamson P. (2000). An evaluation of the combinatorial optimisation approach to the creation of synthetic microdata. Int. J. Population Geography 6, 349–366. doi: 10.1002/1099-1220(200009/10)6:5<349::AID-IJPG196>3.0.CO;2-5

[B28] Wainwright M. J. Jordan M. I. (2008). Graphical Models, Exponential Families, and Variational Inference. Hanover, MA: Now Publishers. doi: 10.1561/9781601981851

[B29] Xu L. Skoularidou M. Cuesta-Infante A. Veeramachaneni K. (2019). “Modeling tabular data using conditional GAN,” in Advances in neural information processing systems (Vancouver, BC: NeurIPS), 32.

[B30] Ye X. Konduri K. Pendyala R. M. Sana B. Waddell P. (2009). “A methodology to match distributions of both household and person attributes in the generation of synthetic populations,” in 88th Annual Meeting of the Transportation Research Board (Washington, DC).

